# Polymer–Based Linear and Symmetric Artificial Synaptic Memristors for Accurate and Reliable Neuromorphic Computing Applications

**DOI:** 10.3390/nano16110657

**Published:** 2026-05-23

**Authors:** Anshu Kumar, Tseung-Yuen Tseng

**Affiliations:** Institute of Electronics, National Yang Ming Chiao Tung University, Hsinchu 30010, Taiwan

**Keywords:** polymer, artificial synapses, potentiation/depression (P/D), memristors, pattern-recognition, neuromorphic computing

## Abstract

The rapid expansion of artificial intelligence has intensified the demand for hardware systems capable of emulating brain-like information processing with high accuracy, energy efficiency, and reliability. Neuromorphic computing based on memristive artificial synapses has emerged as a promising approach to overcome the limitations of conventional von Neumann architectures. Although inorganic and oxide-based synaptic memristors have been widely explored for neuromorphic systems, they often suffer from poor linearity, asymmetric potentiation/depression behavior, limited conductance states, and device variability, which restrict learning accuracy and scalability. In contrast, polymer-based memristors have gained significant attention owing to their intrinsic advantages, including mechanical flexibility, molecular tunability, controllable electronic/ionic transport, low-temperature processability, and compatibility with large-area fabrication. This review critically examines recent advances in polymer—based memristive materials and devices for achieving linear and symmetric artificial synaptic behavior. Polymer synapses are classified into pure polymer, polymer composite, and polymer-hybrid systems through a mechanism to function framework. Rather than providing a general compilation of organic memristor studies, this review analyzes how polymer chemistry, ion-migration control, trap state distribution, redox activity, electrode selection, active layer thickness, and interface engineering govern conductance update linearity, symmetry, and uniformity. Fundamental switching mechanisms, material classifications, device architectures, key synaptic characteristics, and system-level neuromorphic performance, including pattern-recognition applications, are critically discussed. By explicitly linking material and device design to conductance update fidelity, learning accuracy, training convergence, and pattern-recognition reliability, this review provides practical design guidelines and future perspectives for next-generation polymer-based neuromorphic hardware with improved linearity, symmetry, reliability, and scalability.

## 1. Introduction

### 1.1. Neuromorphic Computing Landscape

Neuromorphic computing offers a promising hardware solution to the intrinsic limitations of conventional von Neumann architecture, where the physical separation of memory units and data processing in usual computers leads to severe data-transfer bottlenecks and high energy consumption in artificial intelligence workloads [1,2]. Inspired by the human brain, neuromorphic systems aim to integrate memory and computation at the device level, enabling massively parallel, event—driven information processing with significantly reduced power consumption [3,4]. As neural networks continue to scale in size and complexity, conventional CMOS-based accelerators face fundamental challenges in energy efficiency and memory bandwidth, driving intense interest in material-based neuromorphic hardware [5,6].

Artificial synapses are electronic devices that emulate the function of biological synapses by encoding synaptic weights as continuously tunable conductance states [7,8]. Unlike binary memory elements, artificial synapses rely on analog or multilevel conductance modulation to enable incremental potentiation and depression during learning [9,10]. the key performance metrics of artificial synapses include potentiation/depression (P/D) linearity, P/D symmetry, dynamic range, multilevel resolution, retention, endurance, and energy consumption [11]. Among these, linear and symmetric conductance modulation is particularly critical, as nonlinear or asymmetric weight updates introduce gradient distortion and significantly degrade learning accuracy in neuromorphic systems [12,13].

In neuromorphic training, the calculated gradient is physically implemented as a conductance change in each memristive synapse. Nonlinear P/D modulation makes the update size depend on the current conductance state, causing over-updating in some regions and under-updating near saturation. Asymmetric potentiation and depression further map positive and negative gradients to unequal conductance changes, producing a cumulative update bias. These errors distort the intended gradient-descent trajectory, slow convergence, and reduce pattern-recognition accuracy.

Memristors combine nanoscale size, rapid switching, energy efficiency, and CMOS process compatibility, making them attractive candidates for high–density memory integration and neuromorphic computing applications [14,15]. In general, organic/polymer memristors can be broadly classified into two categories: analog memristors and digital memristors [14]. When functioning as resistive random-access memory (RRAM), these devices demonstrate essential characteristics such as nonvolatile behavior, high storage capacity, rapid switching, low energy usage, a significant ON/OFF resistance ratio, strong endurance, and reliable long—term data retention. In contrast, the latter operates as a logic-like element capable of emulating key synaptic behaviors, including spike-rate-dependent plasticity, spike—timing—dependent plasticity, short- and long-term plasticity, and experience-dependent learning. It thereby demonstrates strong potential for the construction of artificial neural networks (ANNs) in neuromorphic computing systems.

Polymers have attracted considerable interest as artificial synaptic materials owing to their inherent chemical tunability, soft-matter nature, and ability to support both ionic and electronic transport, key properties for emulating biological synaptic behavior in neuromorphic computing systems [16,17,18,19]. In contrast to inorganic oxides, where resistive switching (RS) is often governed by abrupt and stochastic filament formation, polymer matrices enable gradual conductance modulation through homogeneous ion migration, redox reactions, or polaron transport [20,21]. The molecular structure of polymers can be precisely engineered through functional groups, side chains, dopants, and crosslinking to control ion mobility, trap density, and interfacial energetics [22,23,24].

Accurate neuromorphic computing requires artificial synapses to exhibit highly linear and symmetric P/D characteristics to ensure unbiased weight updates and stable training convergence [25,26]. Stable multilevel conductance states with low write noise and minimal drift are essential to preserve learning accuracy during long-term operation [27,28]. In addition, synaptic devices must demonstrate high endurance, sufficient retention, and low programming energy to enable large—scale deployment [29]. Environmental robustness against temperature, humidity, and mechanical stress is increasingly important as neuromorphic hardware moves toward flexible and edge-computing platforms [30]. Polymer-based synapses naturally address many of these requirements, but precise chemical and interface engineering is necessary to minimize variability and nonlinearity [31,32].

This review focuses on polymer-based artificial synapses specifically engineered to achieve linear and symmetric conductance modulation, which is a fundamental requirement for accurate and reliable neuromorphic computing. Although organic memristors have been widely explored for artificial synapses, many reported devices, particularly small-molecule organic systems, organic composites, and hybrid organic switching layers, still suffer from nonlinear conductance modulation, asymmetric potentiation/depression (P/D) behavior, limited stability of intermediate conductance states, large device—to—device and cycle—to cycle—variability, and morphology-dependent switching characteristics [14,16,17,18,19,31,32]. These limitations are commonly associated with uncontrolled charge trapping/detrapping, stochastic conductive-filament formation, nonuniform ion migration, unstable organic/electrode interfaces, and phase separation in the active layer. As a result, synaptic weight updates may become biased, noisy, or poorly reversible, which degrades learning accuracy, retention reliability, and device scalability. Polymer-based systems provide a valuable platform to address these challenges because their backbone structures, side chains, functional groups, dopants, crosslinking density, and polymer–electrode interfaces can be rationally engineered to regulate ionic/electronic transport, trap distribution, redox activity, and mechanical robustness. While previous reviews have broadly covered organic memristors or neuromorphic materials, systematic analyses centered on the chemical and physical origins of linearity and symmetry in polymer synapses remain limited.

Here, we critically examine polymer memristive mechanisms and classify pure polymer, polymer composite, and polymer-hybrid synapses through a mechanism to function framework. Rather than providing a general compilation of organic memristor studies, this review analyzes how polymer chemistry, ion-migration control, trap state distribution, redox activity, electrode selection, active layer thickness, and interface engineering govern linear and symmetric potentiation/depression behavior. By explicitly linking material and device design to conductance-update fidelity, learning accuracy, training convergence, and pattern-recognition reliability, this review provides practical design guidelines for next-generation polymer-based neuromorphic hardware.

### 1.2. Research Interest in Memristors and Polymer-Based Neuromorphic Devices

**Figure 1:**
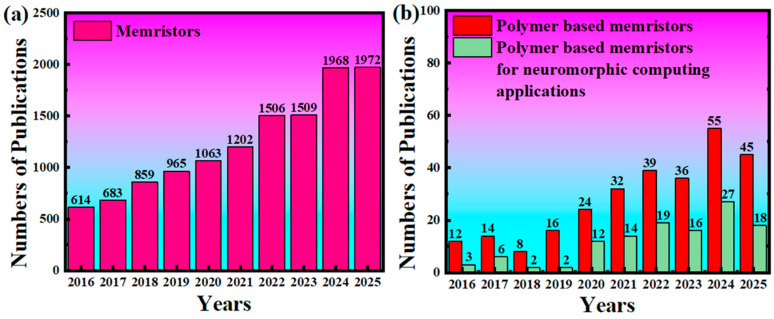
(**a**) Annual growth in the number of publications on memristors from 2016 to 2025. (**b**) Year—wise comparison of publications on polymer—based memristors and their applications in neuromorphic computing over the same period. [Web of science database].

The interest of researchers in memristors has constantly increased over recent years, as evidenced by publication data obtained from the Web of Science database. The number of research publications on memristors shows a clear upward trend, increasing significantly from 2016 to 2025 (Figure 1a), reflecting the growing importance of RS devices in modern computing technologies. This increasing trend is further highlighted in (Figure 1b), where the number of publications on polymer–based memristors has also steadily risen, with an even more pronounced growth in studies focused on neuromorphic computing applications. The continuous increase in polymer-based memristor research indicates a strong shift toward application-driven developments, particularly in artificial synapses and brain-inspired computing systems. The memristive behavior has garnered significant attention owing to its potential applications in low-power devices, non-volatile memory systems, and neuromorphic computing networks. Overall, the observed research trends demonstrate that polymer-based memristors are emerging as a key research direction, with increasing efforts dedicated to developing efficient, scalable, and reliable neuromorphic computing technologies.

## 2. Fundamentals of Polymer Memristive Mechanisms and Selection of Electrode Materials

The performance, reliability, and synaptic accuracy of polymer-based memristors are intrinsically governed by their RS mechanisms. In contrast to inorganic memristive systems, which are typically dominated by abrupt and localized filamentary conduction, polymer memristors operate through chemically tunable and spatially distributed transport processes that enable gradual, analog modulation of conductance. These material—specific mechanisms are central to achieving linear, symmetric, and low variability synaptic weight updates, which are essential for accurate neuromorphic computing. This section outlines the fundamental physical principles underlying polymer memristive behavior, summarizing the dominant ionic and electronic transport pathways and contextualizing them through direct comparison with oxide and perovskite-based memristive systems.

In polymer memristors, switching should be interpreted as a collective ensemble response rather than as an isolated molecular event. Ion hopping, trap occupation, redox conversion, and chain-conformation changes occur within a conformationally disordered soft polymer matrix. In contrast to crystalline oxide memristors, where switching is commonly guided by localized lattice defects, oxygen—vacancy migration paths, or filamentary channels, polymer films contain heterogeneous free volume, variable coordination sites, distributed trap states, and segmental chain motion, which make conductive-path formation intrinsically stochastic. When these microscopic processes are spatially localized, the device exhibits abrupt HRS/LRS transitions and large cycle—to—cycle variability. Conversely, when polymer chemistry, doping concentration, crosslinking density, electrode selection, and interface engineering can be processed to distribute uniformly throughout the active layer of the device, it would lead to that the ensemble conductance evolves gradually, enabling analog conductance modulation with improved linearity and more symmetric P/D updates.

The resistive switching behavior of polymer—based memristors is mainly associated with conductive filament formation/rupture, charge trapping/detrapping, ion migration, conformational change, and charge—transfer processes (Table 1). To explicitly connect these mechanisms with the central requirement of linear and symmetric conductance modulation, each switching pathway should be evaluated according to whether it produces spatially distributed, reversible, and rate-controlled conductance evolution. Ionic migration and redox-controlled doping can provide gradual potentiation/depression (P/D) updates when ion motion is confined and uniformly distributed; however, uncontrolled metal-cation drift, dendritic filament growth, or irreversible redox reactions often produce abrupt switching, nonlinear weight updates, and P/D asymmetry. Charge-transfer and conformational reconfiguration mechanisms are potentially advantageous for linearity because conductance can be modulated through reversible molecular states, but their symmetry strongly depends on balanced donor–acceptor energetics, trap density, and electrode barriers. Charge trapping/detrapping can also support multilevel conductance states when trap sites are uniformly distributed, whereas nonuniform trap populations cause threshold dispersion and abrupt carrier release. Therefore, the design objective for polymer synapses is to convert stochastic localized switching into distributed, reversible, and balanced conductance modulation during both potentiation and depression.

### 2.1. Ionic Migration

**Figure 2:**
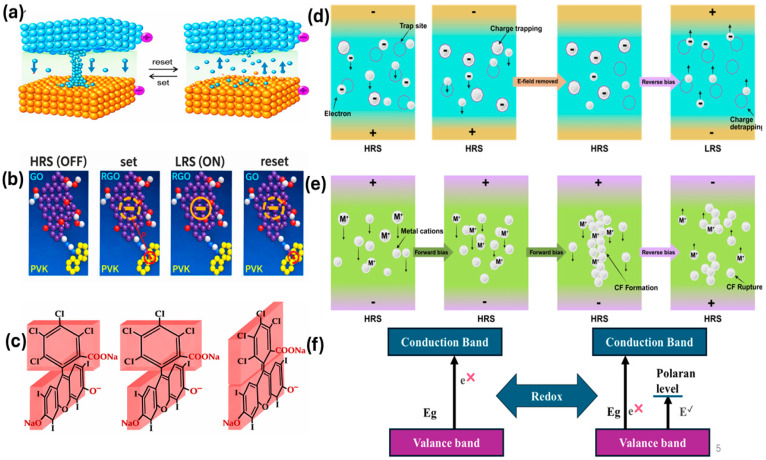
Schematic illustration of the RS mechanisms, including (**a**) ion migration, (**b**) charge transfer, reproduced with permission from ref. [36]. (**c**) Conformational Reconfiguration, reproduced with permission from ref. [36]. (**d**) Charge trapping/de—trapping, reproduced with permission from ref. [36]. (**e**) Conductive filament, reproduced with permission from ref. [37]. (**f**) Redox reaction, reproduced with permission from ref. [36].

Mobile ions within polymer films, serving as charge traps or dopants, can migrate across the insulating layer under an applied electric field [33,34]. For example, when copper (Cu) or silver (Ag) electrodes are utilized in polymer devices, the active metal atoms undergo electrochemical oxidation, generating Cu^2+^ or Ag^+^ ions (Figure 2a). The generated cations are injected from the electrode–polymer interface into the insulating layer, where they drift toward the cathode. Electron injection at the cathode maintains overall electrical neutrality. Depending on the relative mobility of ions and electrons, reduction of metal cations can occur either at the cathode interface in ion—conductive polymers (e.g., polyelectrolytes) or within the polymer bulk in electronically insulating materials. The reduced Ag/Cu atoms nucleate into metallic clusters that progressively grow into conductive filaments (CFs), which eventually bridge the anode and cathode, forming a localized conductive channel and driving the device into the low-resistance state (LRS). The metallic conductive filament can be ruptured under reverse bias through electrochemical oxidation or thermally disrupted by Joule heating under high current, thereby resetting the device to a high-resistance state (HRS). Ion-migration-driven filamentary conduction arises from the coordination and dissociation of metal cations with polymer matrices containing heteroatoms such as oxygen or sulfur, a process facilitated by polymer chain mobility. In addition to extrinsic filament formation via foreign metal ions, the migration of native anions can also induce or stabilize RS. For example, redistribution of PSS^−^ domains in oxidized PEDOT: PSS and perchlorate ions in TPA/viologen bilayer systems results in bistable and memristive switching behavior [35].

### 2.2. Charge Transfer

Charge transfer is a fundamental mechanism underlying RS in organic memristors (Figure 2b). In conjugated polymers and small-molecule systems, this behavior is enabled by rational design of donor—acceptor (D–A) architectures [38,39,40,41]. In donor—acceptor (D–A) systems, an applied electric field drives charge transfer between donor and acceptor units. When the voltage exceeds the turn-on threshold, electron transfer from the donor to the acceptor increases the carrier density, leading to the formation of a conductive pathway between the electrodes. This process switches the device from a HRS to LRS. Upon applying a reverse bias, electron—hole recombination reduces the carrier population, ruptures the conductive pathway, and resets the device from LRS to HRS [41,42]. In D–A systems, the acceptor plays a critical role in charge transfer and charge retention [42,43,44].

### 2.3. Conformational Reconfiguration

The porous architecture of two-dimensional covalent polymer networks provides internal free volume that enables conformational flexibility and structural rearrangement [45,46,47]. Previous studies suggest that the carbazole groups in PVK—PF and iamP6 films are randomly oriented, which disrupts the formation of ordered π—π stacking. This structural disorder hinders charge transport within the conjugated framework, leading to low initial conductivity in pristine polymer devices (Figure 2c). For example, in organic materials, carbazole units regulate charge transport by undergoing conformational rearrangements, which directly induce electrical switching behavior [48]. Under an applied electric field, carbazole units are oxidized to generate positively charged species. These oxidized units interact with adjacent neutral carbazole moieties, driving a conformational reorganization into a face—to—face structure. This conformation enables efficient charge transfer between carbazole units, thereby switching the device to the LRS. The conformation is disrupted under an applied reverse voltage. As a result, the device returns to the HRS. The extent of the conformational change can be controlled by adjusting the compliance current [49].

### 2.4. Charge Trapping/Detrapping

When an external electric field is applied, charge carriers are injected from the electrodes. These carriers enter the resistive—switching layer of the organic memristor. Simultaneously, defects in the material and band-structure inhomogeneity give rise to localized charge traps within the resistive-switching layer [50,51,52]. During the trapping process, charge carriers become confined within localized trap states. This confinement maintains the device in HRS. As the applied voltage increases, trapped charge carriers are released from localized trap states and move into the conductive band or free-carrier states. This process restores the device conductivity, switching it to the LRS (Figure 2d).

### 2.5. Conductive Filament

Conductive filament (CF) formation is widely recognized as the dominant mechanism underlying resistance switching in polymer memristors. This mechanism is the most commonly observed in devices where when a voltage bias is applied, a CF forms within the functional layer. This filament provides a pathway for charge transport. Electrochemical metallization is one of the standard processes used to form a CF. In this process, active metals such as Cu, Ni and Ag act as the anode due to their participation in electrochemical reactions. In contrast, materials like Pt, Au, TiN, and ITO are typically used as cathodes [50,51]. Upon application of a positive voltage, oxidation takes place at the anode interface, generating metal ions. These ions drift toward the cathode under the electric field, where they undergo reduction. The ions are subsequently reduced upon encountering anions at the cathode interface or electrons or within the electrolyte. The accumulation of reduced metal ions results in the formation of CFs between the electrodes, inducing the transition of the memristor from HRS to LRS. Under a negative bias, the CF ruptures via the reverse reaction, thereby returning the memristor to HRS, while polymer materials can serve as functional layers for filament formation (Figure 2e) [53,54].

### 2.6. Redox Reaction

When organic materials are employed as functional layers, an external electric field can induce redox reactions within the material. This results in substantial modulation of the electrical conductivity (Figure 2f) [55,56,57,58,59]. During the oxidation process, the material loses electrons, which results in the formation of positively charged holes or ions. This causes a decrease in the conductivity of the functional layer, producing HRS. Conversely, during the reduction process, the material gains electrons and returns to a neutral or electron-rich state, thereby increasing the conductivity and generating an LRS. Because of these redox characteristics, the memristive behavior of such devices is closely associated with charge trapping and charge transfer processes. Redox reactions can be operated in conjunction with other resistance-switching mechanisms [60,61,62]. For example, in devices based on poly (ethylene oxide) (PEO) doped with CH_3_NH_3_PbBr_3_ nanoparticles (NPs), Pb^2+^ cations generated through electrochemical oxidation at the electrodes interact with the ether oxygen groups on the PEO chains. This interaction enhances the redox activity at the interface layer, promotes the formation of metallic CFs, and ultimately results in resistance-switching behavior [63]. Atanu reported a non-volatile memristor exhibiting artificial synaptic behavior based on redox reactions in organic composites [64]. In addition to the switching mechanisms discussed above, several other mechanisms have also been proposed. These mechanisms aim to explain the switching behavior. These include ohmic conduction, Schottky emission, thermionic emission, Poole–Frenkel emission, and trans-to-cis isomerization [65]. Advanced characterization methods, including in situ microscopy and spectroscopy, provide deeper insight into the switching mechanisms [49].

The advantages and limitations of these mechanisms can be summarized from the perspective of P/D linearity and symmetry. Ion-migration and filamentary mechanisms offer large conductance windows and low operating voltages, but require strict control of ion mobility, filament nucleation sites, and reset kinetics to avoid asymmetric SET/RESET behavior. Charge-transfer, conformational, and redox mechanisms are more compatible with gradual analog modulation because they rely on molecular-state evolution rather than abrupt metallic bridging; nevertheless, saturation of redox centers, slow relaxation, and imbalanced injection barriers may still cause nonlinear conductance evolution. Trap-mediated switching is useful for achieving multiple intermediate states, but only if trap depth, trap density, and spatial distribution are well controlled. These considerations provide the mechanistic basis for the material and architectural strategies discussed in the following sections.

**Table 1:** Reported mechanism based on polymer memristor.

Device Structure	Mechanism	Set/Reset Voltage (V)	ON/OFF Ratio	Ref.
Ag/PVP/Pt	Conductive filament	0.65/−0.49	10^3^	[38]
Al/COF-Azu/ITO	Conformational Reconfiguration	1.95/−0.50	50	[45]
Au/polyimide (PI)/Au	Charge trapping/detrapping	0.6/−0.8	-	[49]
Ag/PEI-AgClO_4_/Pt	Conductive filament	1.2/1.2	10^3^	[53]
Ag/PVA/ITO/PEN	Conductive filament	1.4/−1	10^3^	[66]
Pt/PTEDOT-AuNP/Pt	Charge trapping/detrapping	-	10^3^	[67]
Ag/PCPX-Ag/ITO	Ion migration	-	10^4^	[68]
Ag/EGC-1700/Au/PEN	Conductive filament	1.5/−1	10^5^	[69]
ITO/iamP6/Al	Charge transfer	2/–1.15	10^4^	[70]
ITO/DNOBTDT/Al	Charge transfer	2/–2.76	10^3^	[71]
ITO/P4VPCz5/Al	Conformational change	1/–2.5	10^3^	[72]
ITO\CdSe QDs-PVP\Al	Charge trapping/detrapping	1.6/−1.7	10^5^	[73]
ITO/PAA/PEI/ITO	Ion migration	2.5/1.5	50	[74]
Ag/Ag-doped PVA/Pt	Conductive filament	1.0/−0.6	10^5^	[75]
Ag/2DPBTA + PDA/ITO	Conductive filament	0.90/−1	10^5^	[76]
Ag/ZnO NS@PMMA/Pt	Charge trapping/detrapping	0.4/−0.4	5	[77]
Al/WS2:PMMA/ITO/PEN	Charge trapping/detrapping	0.5/−4.4 V	6 × 10^4^	[78]
Ag/borophene-PVA nanofibers/ITO	Conductive filament	0.95 V/–1.33 V	4.1 × 10^3^	[79]

### 2.7. Electrode Materials

Memristor electrodes provide pathways for electric current. They also actively contribute to the process. They are typically fabricated from a wide range of materials. Electrode materials are generally categorized into four main classes. This classification is defined according to their roles in resistive switching behavior. First, the electrodes are typically composed of inert metals (e.g., Au, Pt). Second, they can directly contribute to CF formation, as observed in cation migration-driven polymer memristors. In these memristors, CFs arise from electrochemical dissolution and subsequent metal deposition, predominantly involving Cu and Ag. Finally, a range of emerging electrode materials has been explored, including ITO FTO and graphene, which are widely employed in flexible and transparent polymer memristors [74].

Electrode selection is particularly important for linear and symmetric artificial synapses because it determines carrier injection barriers, ion supply, and the polarity dependence of switching. Symmetric inert electrodes can balance injection and extraction processes, thereby improving P/D symmetry, whereas active electrodes such as Ag or Cu can lower the SET voltage and increase the dynamic range but may also create excessive ion injection and abrupt conductive-filament formation. Thus, the electrode pair should be chosen not only for low resistance or process compatibility, but also for balanced potentiation/depression kinetics and reduced cycle-to-cycle variation.

### 2.8. Effect of Electrode Materials

When a bias voltage is applied between the electrodes, a current pathway is established between the top and bottom electrodes (BEs) of the memristor. Even with the same active layer, devices can exhibit different electrical properties. This occurs when different top and BEs are used. This section explores the effect of electrode materials on the performance of memristor devices.

Ree et al. developed memristor devices based on poly (o-anthranilic acid) as the active switching layer. In these devices, Au and Al functioned as the top electrodes (TEs), whereas ITO and Au were employed as the BEs [80]. With ITO as the BE and Au as the TE, the device exhibits a V_off_ of +1.60 V and V_on_ of −0.5 V. The ON/OFF current ratio is 1.0 × 10^4^. Changing the top electrode from Au to Al shifts V_off_ from +1.60 to +1.90 V and V_on_ from −0.5 to −0.75 V, while also lowering the ON/OFF ratio to 1.5 × 10^3^. These differences in switching voltages are primarily due to the asymmetric electrode arrangement. The use of symmetric electrodes (Au/Au) results in markedly better electrical performance than asymmetric configurations (Au/ITO or Al/ITO). The switching voltages are nearly identical, with V_off_ of +0.84 V and V_on_ of −0.87 V. This symmetry is advantageous, as it simplifies control circuit design and enhances operational efficiency in memristors. Additionally, the maximum ON/OFF ratio exceeds 1 × 10^5^ in devices with Au–Au electrodes.

Ouyang first reported an electrode-sensitive RS device with a TE/PS + Au-2NT/Al configuration (TE: Cu, Au). In this system, the active layer comprised a polystyrene (PS) matrix incorporating Au–2-naphthalenethiol (2NT) NPs [81]. The Au/PS + Au–2NT/Al device maintains its OFF state over a voltage sweep ranging from 0 to 2.5 V. By contrast, sweeping the voltage from 0 to −2.5 V drives the device into the ON state, where the current density |J| rises sharply from 1.0 × 10^−3^ to 2.3 × 10^−2^ A cm^−2^ (at 1.0 V). During a reverse sweep (0 → −2.5 V → 0 V), the device returns to the OFF state. The OFF-to-ON transition occurs at a threshold voltage (Vth) of −2.0 V, reaching a maximum |J| of 0.98 A cm^−2^. A clear difference in device performance is observed when Au is replaced by Cu as the top electrode. During a negatively biased sweep (0 → −3.0 V → 0 V), the device transitions from OFF to ON and exhibits bipolar switching, with the erasing process occurring under positive bias. Replacing the Au top electrode results in a shift of V_on_ from −2.0 to −2.3 V, along with a substantial increase in the maximum |J| to 6.8 A cm^−2^. The differences in switching behavior originate from the varying work functions of the metal electrodes, which are approximately 4.6 eV for Cu, 4.1 eV for Al, and 5.1 eV for Au. Applying a positive bias to the Al (BE) electrode in the Au/PS + Au-2NT/Al device causes electrons to be injected from Al into the Au-2NT nanoparticles (NPs), from where they migrate toward either the Cu or Al electrode. However, the greater work function mismatch between Au-2NT and Cu, compared to that of Al and Au-2NT, facilitates more efficient electron injection from Cu into Au-2NT, resulting in a higher programming voltage and an increased ON-state current. As a result, NPs close to the electrode become negatively charged. In contrast, no electron transfer occurs between the Au-2NT NPs and the Au electrode. With a positive bias applied, the electric field at the Al/Au-2NT nanoparticle interface is directed opposite to the external field. This external field is directed from the Al electrode to the Au-2NT NPs, thereby keeping the device in the HRS. Applying a negative voltage eliminates the internal electric field. Consequently, the device transitions into the low-resistance state. Similarly, in the Cu/PS + Au-2NT/Al device, a comparable internal electric field can develop. This field forms between the Au-2NT NPs.

Because of their outstanding mechanical, electrical, and optical properties, graphene and its derivatives have attracted significant attention for use in electronic and energy devices.

Reduced graphene oxide (rGO) thin films exhibit favorable electrical conductivity. This makes them promising candidates for use as electrode materials in memristor devices. The influence of rGO film sheet resistance on ON/OFF currents, switching voltage, and the ON/OFF ratio was systematically studied. This investigation was conducted using rGO/P3HT: PCBM/Al device structures. As the sheet resistance of the rGO films increases from 0.3 to 2.4 and 10.0 kΩ sq^−1^, the ON-state current decreases from 2.2 × 10^−4^ to 7.3 × 10^−5^ and 6.2 × 10^−5^ A. At the same time, the ON/OFF ratio drops from 4 × 10^5^ to 2.2 × 10^4^ and 1.2 × 10^4^. In contrast, the OFF-state current rises from 5.7 × 10^−10^ to 2.8 × 10^−9^ and 5.1 × 10^−9^ A. The switching voltage gradually increases from 0.5 to 0.9 and then to 1.2 V. It is suggested that an increase in the sheet resistance of rGO films suppresses the rate of carrier injection into the polymer active layer. As a result, the switching Vth increases while the OFF-state current decreases.

### 2.9. Effect of Active Layer Thickness

The thickness of the active layer critically influences the RS behavior of polymer memristors. When a bias voltage is applied, the internal electric field depends on the thickness of the active layer, thereby influencing the device’s switching behavior and conduction characteristics. For example, Ouyang fabricated Al/PS + Au-2NT NP/Au devices with polymer thicknesses of 34, 70, and 90 nm. All of these devices exhibit clear RS behavior [81]. However, thinner polymer layers result in significantly higher ON/OFF ratios. For instance, the 34 nm device exhibits a ratio of 38, whereas the 90 nm device shows a ratio of 9.3 (read at −0.5 V). This behavior can be explained in terms of the fundamental relationship between the applied voltage (V) and current density (J) across the device:

(1) $$J=\frac{V}{\left(R_{f}+R_{c}\right)A}$$

where $R_{f}$ is the resistance of the polymer: NP film, A denotes the device area and $R_{c}$ corresponds to the contact resistance at the interface between the electrodes and organic active layer. The programming voltage ($V_{\mathrm{th}}$) shows a weak dependence on the active layer thickness (t):

(2) $$V_{th}=1.76+1.39\times 10^{-3}t$$

The first term (1.76 V) represents the initial voltage drop at *t* = 0, indicating an inherent material property. The second term corresponds to the voltage drop within the active layer, which depends on its thickness. According to this relation, increasing the active layer thickness by 100 nm results in a voltage drop increase of 0.139 V.

From the viewpoint of linearity and symmetry, active layer thickness and interfacial quality determine the internal electric field distribution and the probability of localized hot spot formation. Ultrathin layers reduce operating voltage but can promote abrupt tunneling or filamentary shorting, whereas overly thick films require higher fields and may generate spatially nonuniform ion migration. An optimized thickness therefore supports gradual conductance modulation by distributing the electric field and balancing SET and RESET processes across repeated pulse operation.

Having discussed the key switching mechanisms, electrode effects, and active-layer thickness dependence, the next step is to classify polymer-based memristors according to their material composition and structural design. Such classification is essential because different polymer systems, including pristine polymers, polymer–ion composites, polymer–nanoparticle hybrids, heterojunctions, and two-dimensional conjugated polymers, exhibit distinct charge-transport pathways and synaptic characteristics.

## 3. Classification of Polymer Memristor

### 3.1. Polymer-Based Memristors

**Figure 3:**
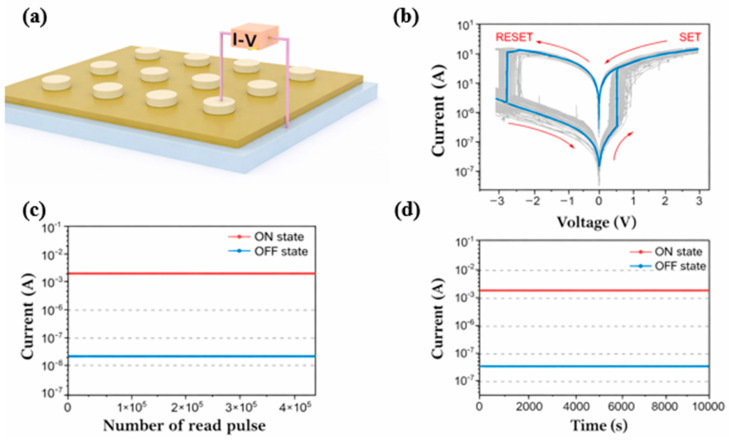
(**a**) Schematic illustration of the polymer memristor device structure, (**b**) I–V characteristic of the memristor, (**c**) effect of continuous read pulses of 0.1 V on the ON and OFF state currents of memristor; pulse width = 1 μS; pulse period = 2.2 μS, (**d**) retention of the polymer memristor device. Reuse with permission from ref. [83].

Organic polymer memristors have attracted increasing attention owing to the intrinsic structural versatility and tunable functionality of polymer materials. The performance of organic polymer memristors is strongly governed by their molecular structure, particularly the architecture of the polymer backbone and the nature of the side-chain functional groups [36]. By systematically adjusting polymer chain topology, functional group distribution, and molecular weight, charge transport pathways and interfacial behaviors can be precisely controlled at the molecular scale. These structural features directly determine the memristive characteristics of the device. For example, Zhang et al. developed a redox-modified conjugated polymer memristive device based on PFTPA-Fc, where redox active moieties (triphenylamine and ferrocene) were introduced onto a fluorene backbone [48]. The device structure consisted of an ITO BE, a thin PFTPA-Fc active layer, and a top metal contact. This polymer memristor exhibited stable RS with multiple conductance levels and could perform basic arithmetic and logic functions through programmable conductance states. The switching was attributed to solid-state electrochemical redox processes of the pendant groups, which modulated charge transport in the polymer backbone [48]. Zhang et al. developed a redox coregulated cathodic electrosynthesis and molecular-potential method for ionic azulene-based thin films in organic memristors. The fabricated Al/PPMAz—Py^+^Br^−^/ITO devices demonstrate a high ON/OFF current ratio of 1.8 × 10^3^, along with excellent stability, long retention time, and strong endurance over a wide voltage sweep range (Figure 3a–d). Notably, the devices also demonstrate distinct multilevel switching behavior and pronounced history-dependent memristive characteristics [82].

For pristine polymer memristors, the principal advantage is molecular–level tunability: backbone conjugation, side-chain polarity, redox groups, and molecular packing can be engineered to regulate charge transport and produce gradual conductance changes. However, pure polymers may also suffer from limited mobile ion reservoirs, trap inhomogeneity, and redox-state saturation, which can restrict the available conductance window or introduce nonlinear P/D behavior. Achieving symmetric modulation in this class therefore requires careful matching of polymer energetics with electrode work functions and control of defect/trap distributions within the active layer. Polymer–based memristors show low operating voltage, good retention, reasonable endurance, and competitive switching ratios. Table 2 compares the device performance of polymer–based memristors with perovskite and oxide memristors. Although some oxide and perovskite devices exhibit higher endurance or retention, polymer memristors offer clear advantages in flexibility, solution processability, low-cost fabrication, and tunable switching behavior, making them promising candidates for neuromorphic applications.

### 3.2. Polymer—Metal—Ion Composite Memristors

**Figure 4:**
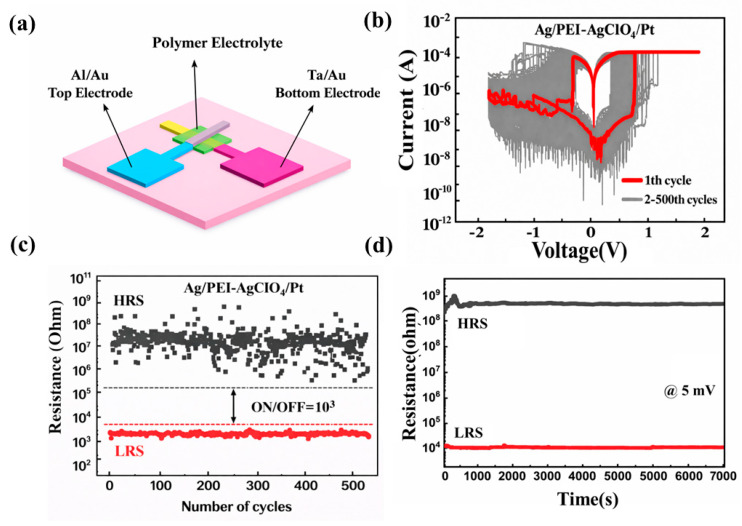
(**a**) Schematic illustration of the polymer metal-ion composites device structure, (**b**) I–V characteristic of the memristor, (**c**) endurance of the polymer metal-ion composites device, (**d**) retention of the polymer metal-ion composite device. Reuse with permission from ref. [53].

In particular, polymer-based memristors have attracted considerable attention for flexible electronic applications due to their flexibility and excellent mechanical stretchability, low fabrication cost, intrinsic viscoelasticity, and good thermal stability [53,55,82,83,84,85,86,87,88,89,90,91]. Despite these advances, most polymer memristors still exhibit poor reproducibility, limited operational stability, and insufficient thermal tolerance, which severely impede their practical application. To overcome these limitations, polymer–ion composite strategies have been widely explored. In this context, it has been demonstrated that Ag-ion doping in polyethylenimine (PEI) enables the realization of robust nonvolatile memristors with thermally stable RS behavior. The PEI features a rigid six-membered ring-based molecular framework. The presence of abundant neutral and charged amine groups enables strong interchain hydrogen bonding, thereby enhancing intermolecular cohesion and significantly improving the thermal stability of the polymer [52,69,88]. Zhang et al. developed a flexible polymer memristor using PEI in combination with an Ag salt as the active layer. Compared to the salt–free device, the incorporation of the Ag salt supplies mobile Ag^+^ ions, which significantly facilitates the formation of CFs and enhances RS behavior. To further evaluate the performance of flexible electronic devices, Zhang et al. fabricated memristive devices on flexible substrates. The device architecture was Ag/PEI—AgClO_4_/Pt Figure 4a. The RS behavior of the memristor was systematically evaluated. The device maintains stable switching characteristics under bending radii of 5, 7.5, and 10 mm, exhibiting a low SET voltage (~1.5 V) and a high ON/OFF current ratio (~10^4^), but unstable operational voltage as shown in Figure 4b. The memristor maintains good RS behavior after 200 bending cycles. This study demonstrates an effective strategy for enhancing the RS performance of polymer-based memristors. As shown in Figure 4c,d the memristor device demonstrated outstanding performance at room temperature, characterized by good endurance and reliable retention.

For polymer–metal-ion composites, the main benefit for linearity is that mobile ions can be deliberately introduced and regulated by polymer coordination sites, enabling progressive conductive-path formation and multiple intermediate conductance states. At the same time, excessive ion concentration or weak coordination can accelerate dendritic filament growth and cause abrupt SET transitions, large reset variability, and asymmetric P/D updates. Consequently, ion concentration, polymer–ion binding strength, and active-electrode reactivity must be optimized to obtain gradual and balanced conductance evolution.

### 3.3. Polymer—Nanoparticle Hybrid Memristors

**Figure 5:**
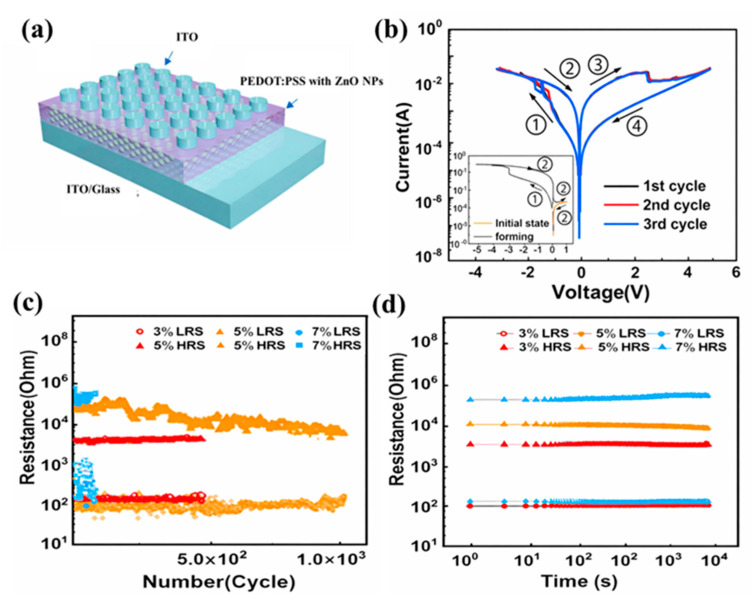
(**a**) Schematic illustration of the polymer–nanoparticle hybrid memristor device structure, (**b**) I–V characteristic of the memristor, (**c**) endurance of the polymer—nanoparticle hybrid memristor, (**d**) retention of the polymer–nanoparticle hybrid memristor device. Reuse with permission from ref. [98].

In recent years, hybrid composite materials formed by integrating polymers with NPs have been extensively developed. These composite systems provide key advantages, including processability, cost-effectiveness, mechanical flexibility, and chemically tunable synthesis, while their RS characteristics can be systematically tuned by varying the NP concentration. A wide range of preparation strategies has been developed for organic nanocomposite materials, with commonly employed methods including solution blending, sol–gel processing, in situ polymerization, hydrothermal synthesis method, and electrochemical deposition [92,93,94,95,96,97]. Among these approaches, solution blending is the most widely adopted method for fabricating the functional layers of nanocomposite memristors. Jiang et al. fabricated polymer–NP hybrid memristors by introducing ZnO NPs into a PEDOT: PSS functional layer and depositing the composite via spin coating to form ITO/PEDOT: PSS (ZnO NPs)/ITO devices (Figure 5a). By tuning the ZnO NP concentration, the devices exhibited optimized RS and synaptic functionality, demonstrating concentration-dependent modulation of switching behavior in polymer nanocomposites (Figure 5b–d) [98]. In addition, Jiang et al. synthesized a hybrid nanocomposite by embedding CsPbBr_3_ perovskite quantum dots into a block copolymer (polystyrene-poly (2-vinyl pyridine)) and fabricated CsPbBr_3_-polymer composite films using spin—coating. The resulting memristive devices exhibited robust RS and negative differential resistance behavior with exceptional stability over 5000 switching cycles and long-term retention. They demonstrated light-tunable memory performance suitable for optoelectronic and neuromorphic applications [99].

The performance of organic nanocomposite memristors is fundamentally governed by the synergistic coupling between the polymer matrix and the incorporated NPs. This enhancement primarily arises from the quantum confinement effects and abundant surface-active sites of the NPs, which enable precise regulation of charge trapping and transport. Simultaneously, interfacial interactions between the NPs and the polymer matrix facilitate the formation of conductive pathways and promote dynamic modulation of charge transport [100,101,102,103].

For polymer nanoparticle hybrids, nanoparticles provide tunable trap sites, local field modulation, and charge reservoirs that can smooth conductance evolution and enlarge the number of accessible synaptic states. Their challenge is that nanoparticle aggregation, percolation-path formation, and interfacial defects can generate localized switching hot spots, leading to nonlinear or asymmetric potentiation/depression. Uniform nanoparticle dispersion and controlled polymer–nanoparticle interfacial coupling are therefore essential for converting nanocomposite switching into reliable linear analog updates.

### 3.4. Polymer Heterojunction—Based Memristors

A heterojunction is an interfacial region formed at the contact between two dissimilar materials [104]. Such interfaces exhibit a distinct set of physical and chemical characteristics that fundamentally differ from those of homojunctions formed between identical materials. The rational structural design of heterojunction-based devices offers new opportunities and advantageous features for the development of high—performance memristor devices [15,105,106,107,108]. In heterojunction memristor devices, the organic heterojunction layer functions as an intermediate active layer with tunable electrical conductivity. Zhang et al. developed a low-power artificial synapse device based on the P3HT: PCBM heterojunction, reducing power consumption by 30 times compared to single-layer poly (3 hexylthiophene–2,5-diyl) (P3HT) or [6]-phenyl-C61-butyric acid methyl ester (PCBM) through solid solution processing and thermal treatment. This device successfully simulates multidimensional synaptic plasticity—spike rate (SRDP)-, amplitude (SADP)-, pulse width (SDDP)-, and timing (STDP)-dependent characteristics—and performs better than most inorganic devices in high-temperature environments. In addition, Luo et al. built a PEDOT: PSS/pentacene heterojunction where the thin pentacene layer tunes the injection barrier, enabling low-voltage analog RS and stable synaptic plasticity under mechanical bending [108].

For polymer heterojunction memristors, the interface can be used as an additional design parameter to tune injection barriers, separate ionic and electronic transport, and stabilize gradual conductance modulation. Properly engineered heterojunctions can reduce programming voltage and improve P/D symmetry by balancing carrier injection during opposite pulse polarities. However, interfacial roughness, energy-level mismatch, phase separation, or mechanically induced delamination may create asymmetric barriers and nonuniform switching, so interfacial stability is a critical requirement for linear synaptic operation.

### 3.5. 2D Conjugated Polymer–Based Memristors

**Figure 6:**
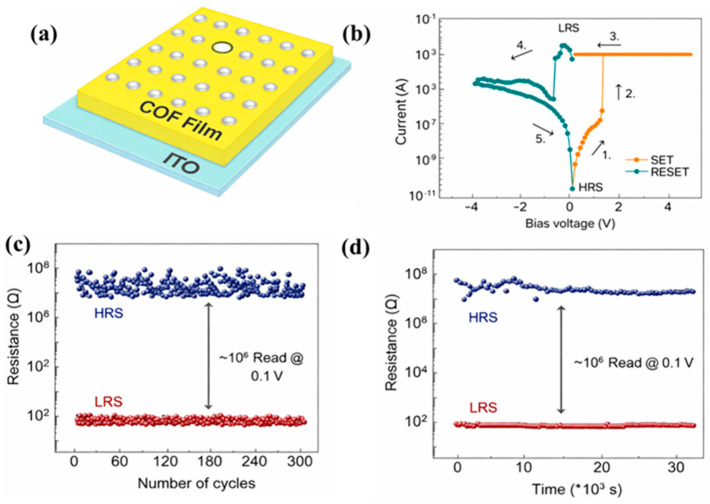
(**a**) Schematic illustration of the 2D conjugated polymer—based memristors device structure, (**b**) I–V characteristic of the memristor, (**c**) endurance of the 2D conjugated polymer-based memristor, (**d**) retention of the 2D conjugated polymer-based memristor device. Reuse with permission from ref. [112].

Two-dimensional covalent polymer (2DCP) thin films are promising materials for next-generation resistive memory devices. This is due to their tunable electronic properties and mechanical flexibility, high uniformity, well-defined pore structures, strong structural stability and low density [109]. 2DCP–based memristors generally employ a metal insulator metal (MIM) architecture. This structure enables reversible switching between HRS and LRS states, characteristic of nonvolatile memory devices. Donor–acceptor (D–A) architectures intrinsically enable electric-field-driven charge separation and carrier transport, thereby imparting inherent RS functionality to 2DCP materials. Liu and co-workers reported a donor–acceptor (D–A) 2DCP, denoted as PI-NT, fabricated as a thin film exhibiting high crystallinity, well-defined molecular orientation, tunable thickness, and low surface roughness. The polymer framework was constructed from electron-donor triphenylamine units and electron—acceptor naphthalene diimide units. The PI-NT-based two-terminal sandwich device, incorporating a LiF interlayer and Al electrodes, demonstrates typical nonvolatile write-once–read-many (WORM) behaviors with threshold voltages of +2.30 and −2.64 V, under both positive and negative voltage sweeps, respectively. It achieves an ON/OFF current ratio above 10^6^ under positive bias and in the range of 10^4^–10^6^ under negative bias, along with excellent operational stability and long-term data retention. The observed RS is mainly driven by an electric-field-induced charge-transfer process. This mechanism is further strengthened by efficient intramolecular charge transfer and the stabilization of charge-separated states within the donor–acceptor (D–A) framework. Furthermore, Yu and co-workers designed an imine-linked two-dimensional covalent polymer (2D CP), termed COF-TT-BT, by integrating electron–deficient triazine units with electron-rich thiophene moieties. This donor–acceptor (D–A) architecture enables high-performance and reliable rewritable memristor operation (Figure 6a). The memristors based on COF–TT–BT demonstrate well-defined bipolar I–V behavior with a low operating voltage of 1.30 V (Figure 6b) [110]. In addition, under a low bias of 0.1 V, memristors based on COF-TT-BT maintain stable switching over 319 cycles and exhibit a retention time of up to 3.3 × 10^4^ s (Figure 6c,d) [111]. Chen and co-workers reported the fabrication of an azu–based 2DCP thin film (COF-Azu) via a liquid–liquid interfacial polymerization method for high-performance memristor applications.

The Al/COF-Azu/ITO devices demonstrate nonvolatile RS with an SET voltage of 0.50 V and a data retention time up to 3 × 10^4^ s. Under a pulse train of ±0.1 V amplitude, 50 ms pulse width, and 20 ms interval, the device shows gradual current modulation, closely mimicking long-term potentiation and depression in synaptic systems. When identical pulse trains (pulse width: 20 ms) are applied to memristor cells within an array, pronounced analog SET and RESET behaviors are observed. Exploiting these memristive features, a convolutional neural network was applied to image recognition, reaching an accuracy of 80% after eight training epochs [42]. Hu and co-workers employed terephthalaldehyde derivatives bearing alkoxy chains of varying lengths to synthesize ultrathin two-dimensional covalent polymers (2DCPs; 2DPTAPB + TPOC) with single-monomer thickness via the Langmuir Blodgett technique. Their study showed that extending the alkoxy chain length promotes the formation of highly crystalline films while reducing metal penetration during thermal evaporation of the top electrode. Consequently, the fabricated Ag/2DPTAPB + TPOC/ITO devices exhibit high operational reliability (>300 consecutive cycles), low switching variability (σVset = 0.14), an ultralow operating voltage of 0.6 V and excellent stability (>10^5^ s). Notably, the ultrathin memristors also show exceptional mechanical robustness, maintaining stable performance under bending strains of up to 2.6% [112].

For 2D conjugated polymer systems, long-range order, well-defined pore structures, and oriented donor acceptor frameworks are advantageous for achieving uniform charge transport and reduced switching variability. These structural features can support more reproducible analog SET/RESET processes and therefore improve both linearity and symmetry. The remaining challenges are controlling film thickness, crystallographic orientation, grain boundaries, and top–electrode penetration during fabrication, since defects in ultrathin 2D polymer layers can still produce localized current paths and disturb balanced P/D modulation.

**Table 2:** Comparison of polymer-based memristor with different types of memristors.

Configuration	Structure	Set/Reset Voltage	Endurance (Cycles)	Retention (s)	Switching Ratio	Ref.
Polymer memristor	Ag/PVA/ITO	1.4/–1	3 × 10^2^	6 × 10^3^	10^4^	[67]
	Ag/2DP_BTA+PDA_/ITO	1.5/–3.3	2 × 10^2^	3.5 × 10^4^	>10^5^	[74]
	Ag/2DPBTA + PDA/ITO	0.90/−1	2 × 10^2^	8 × 10^4^	10^5^	[76]
	Ag/PEI/Pt	1/–0.8	10^3^	≈10^5^	≈10^5^	[84]
	Ag/SU-8Ag/Pt	0.3/0.7	10^2^	2 × 10^3^	10^6^	[113]
	ITO/PVA-GO/ITO	−0.2/0.2	5 × 10^2^	10^4^	>10	[114]
	Au/2DPTPAK + TAPB/ITO	1.26/-	10^3^	20−40	10^2^	[115]
	Ag/PVK: TCNQ/ITO	0.5/–0.2	10^4^	10^4^	10^3^	[116]
Perovskite memristor	Au/Cs_2_AgBiBr_6_/ITO	1.5/−3.4	10^2^	10^5^	>10	[117]
	Au/MASnBr_3_/ITO	0.6/−3.5	10^4^	10^4^	10–10^3^	[118]
	Au/Cs_3_Bi_2_I_9_/ITO	0.3/−0.5	10^2^	10^4^	10^3^	[119]
	ITO/Ag/MAPbI3/Au	2.4/−2.2	~10^6^	4.2 × 10^7^	~10^7^	[120]
Oxide memristor	ITO/ZTO/MgO/ITO	0.55/−1.7	10^3^	10^4^	10^2^	[121]
	Pt/Ta_2_O_5−x_/TaO_2−x_/Pt	−1/2	10^12^	-	>10	[122]

The above classification highlights how material composition and molecular architecture determine the resistive switching behavior of polymer memristors. However, the practical performance of these materials also strongly depends on device configuration and fabrication strategy. Therefore, the following section focuses on device architectures and processing methods that translate material-level advantages into reliable memristive and synaptic device operation.

## 4. Device Architectures and Fabrication Strategies

Device architecture determines how the electric field, ionic flux, and current density are distributed across the polymer active layer. Therefore, architecture directly affects whether conductance modulation proceeds gradually and symmetrically or through localized, abrupt switching. The following subsections clarify the role of two-terminal, vertical, planar, and fabrication-related design choices in promoting improved linearity and P/D symmetry.

### 4.1. Two-Terminal Memristors

Two-terminal memristors typically utilize a MIM configuration. In this structure, an active functional layer is sandwiched between two electrodes, allowing electrically driven and reversible modulation of the device resistance. The resistance state of a memristor is modulated by the applied electrical stimulus, enabling information storage through stable and distinguishable resistance levels within a single device. The RS behavior arises from controlled, electric-field-induced physical reconfiguration of the active layer, allowing reversible transitions between at least two distinct conductance states [123]. When implemented as artificial synapses, memristors offer intrinsic advantages including nanoscale device dimensions, structurally simple architectures, and high compatibility with conventional microfabrication processes, while emulating synaptic plasticity through programmable and gradual modulation of conductance. From an information-transmission perspective, the stimulus–dependent evolution of memristor conductance closely mirrors the activity-driven adjustment of synaptic weights in biological neural systems [124,125,126]. Owing to its structural simplicity and distinctive operating mechanisms, memristor development has progressed in parallel with the rapid growth of neuromorphic device research since 2008.

For linear and symmetric conductance modulation, the key advantage of the two-terminal geometry is its direct mapping between device conductance and synaptic weight, which simplifies array integration and learning implementation. The main challenge is that the same two electrodes simultaneously control readout, ion injection, and switching, so asymmetric electrode reactions or localized field enhancement can easily distort potentiation and depression trajectories. Balanced electrode selection, current compliance, pulse engineering, and interfacial buffer layers are therefore needed to obtain analog rather than binary switching.

### 4.2. Vertical of the Two-Terminal Structure

**Figure 7:**
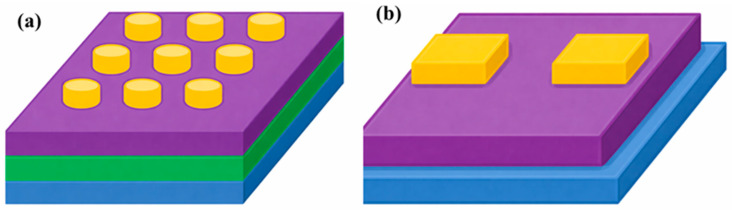
Schematic illustration of two-terminal memristors, (**a**) vertical structure, (**b**) plane structure.

The vertical MIM, also known as a sandwich-type configuration, is the most widely employed structure in polymer memristive devices, where the active resistive-switching polymer layer is positioned between the top and BE. Figure 7a [82]. Liu et al. fabricated an organic two-terminal memristive device for neuromorphic computing, in which a semicrystalline block copolymer, poly(butylene furandicarboxylate)-b-(ε-caprolactone) (PBFCL), was spin-coated onto an Ag/SiO_2_/Si substrate to function as the active channel layer [127]. The high density of polar functional groups, together with the pronounced π–π ordering of the rigid furan chromophores, effectively regulates metal conductive filament formation and charge transport within the device. A device feature size of 50 nm and compact 32 × 32 integrated matrices based on an organic semiconductor were successfully achieved. Organic materials offer distinctive advantages in photo responsive behavior compared with the metal-oxide memristors that dominate current technologies; consequently, optical stimuli are frequently employed as effective inputs to enable multidimensional neuromorphic responses in two-terminal devices. Wang et al. synthesized a narrow-bandgap polymer (P1) featuring a donor–acceptor–donor–acceptor′ (D–A–D–A′) architecture, incorporating 4,4-bis(2-ethylhexyl)-4H-cyclopenta [2,1-b:3,4-b′]dithiophene (CPDT),benzo [1,2-c;4,5-c′]bis [1,2,5]thiadiazole (BBT), and (E)-4,4′-bis(2-octyldodecyl)-[6,6′-bithieno [3,2-b]pyrrolylidene]-5,5′(4H,4′H)-dione (TIG) moieties. Poly(3-hexylthiophene) [127].

Vertical structures are advantageous for high-density arrays because the short vertical transport path lowers voltage and supports compact crossbar integration. However, the strong out-of-plane electric field can concentrate ion migration or filament growth at nanoscale defects, resulting in abrupt conductance jumps and device–to–device variability. Linear and symmetric operation in vertical polymer memristors therefore requires uniform film thickness, smooth electrodes, controlled active-electrode dissolution, and interlayers that distribute ion reduction/oxidation events throughout the active layer.

### 4.3. Plane of the Two Terminal Structure

Planar architectures position the functional polymer layer laterally between two electrodes on the same substrate. Lu et al. reported lateral two-terminal memristors based on pentacene thin films fabricated on Si/SiO_2_ and flexible polyethylene terephthalate (PET) substrates. The planar devices exhibited both digital and analog RS behaviors, along with a rich set of synaptic plasticity functions that could be effectively modulated by varying electrical stimulation protocols—see Figure 7b [128].

Planar structures provide direct access to the active channel and are useful for visualizing switching pathways, mechanically flexible layouts, and optoelectronic modulation. Because the lateral electrode spacing can be designed independently, planar devices may reduce vertical shorting and allow more gradual modulation over a longer channel length. Nevertheless, large lateral gaps increase operating voltage, and nonuniform edge fields near the electrodes can still induce asymmetric conductance evolution. Electrode geometry, channel length, and surface morphology must therefore be optimized to balance potentiation and depression kinetics.

### 4.4. Fabrication Strategies of Polymer Memristor

Compared with alternative fabrication approaches, solution processing offers significant advantages, including operational simplicity, cost–effectiveness, and broad compatibility with a wide range of precursor materials [129]. Polymer–based memristors can be readily fabricated using solution-processable methods such as dip coating, spray coating, spin coating, roll–to–roll coating, and inkjet printing. These methods enable deposition on a broad variety of substrates, including metal foils, glass and flexible plastics [130]. Among these methods, spin coating is the most widely employed, as it enables the formation of uniform, high-quality thin films from solution while offering good process compatibility and reproducibility [131]. By tuning the spin speed and duration, the film thickness and uniformity can be precisely controlled, simplifying fabrication and improving the performance and reliability of heterojunction memristors [132]. Zhang et al. fabricated P3OT-based organic polymer memristors using a simple solution spin-coating route in a vertical ITO/P3OT/Al architecture. The devices exhibited typical memristive behavior with high conductivity and stability [101]. In addition, Zhang et al. fabricated polymer memristors based on a two-dimensional conjugated polymer using a solution spin-coating deposition method in a vertical metal/polymer/ITO architecture, which was further scaled into crossbar arrays. The devices exhibit uniform and stable memristive switching, with a low operating voltage (~±0.3 V) and a high ON/OFF ratio (~10^3^). They also show minimal device-to-device variation and an excellent production yield approaching 90%.

Fabrication strategy is also directly linked to linearity and symmetry because film roughness, residual solvent, crystallinity, dopant distribution, nanoparticle dispersion, and electrode damage all influence local electric fields and switching site statistics. Spin coating offers good laboratory-scale uniformity, while printing and roll–to–roll processes are attractive for scalable flexible electronics; however, scalable methods must tightly control thickness variation and drying-induced phase separation to avoid large device-to-device variation. Process optimization should therefore be treated as a synaptic-performance parameter, not only as a manufacturing consideration, because uniform active layers and clean interfaces are prerequisites for reproducible linear and symmetric P/D updates.

Although device architecture and fabrication methods determine structural uniformity and scalability, accurate neuromorphic computing further requires precise control over conductance modulation. In particular, linear and symmetric potentiation/depression behavior is essential for minimizing weight–update errors and improving learning accuracy. The next section therefore discusses strategies for achieving linear and symmetric synaptic conductance in polymer-based memristors.

## 5. Achieving Linear and Symmetric Synaptic Conductance

Polymer-based memristors have emerged as highly attractive alternatives to conventional inorganic memristors, owing to their low-cost and scalable fabrication, mechanical flexibility, facile processability, intrinsic biocompatibility, and the ability to precisely tailor optical and electrical properties through rational molecular and structural design [17,20,133]. These characteristics enable polymer memristors to meet the stringent mechanical adaptability and functional requirements of flexible neuromorphic electronics and wearable artificial intelligence devices. Nevertheless, despite substantial advances, state-of-the-art two-terminal memristors continue to face fundamental challenges that hinder their practical deployment in large-scale neuromorphic networks. Key technical limitations include pronounced device-to-device and cycle-to-cycle variability in RS, limited controllability over analog conductance modulation, and strongly nonlinear potentiation and depression behaviors [133].

Such nonlinear characteristics severely degrade neural network accuracy and learning efficiency, in stark contrast to biological synapses, which exhibit near-linear and symmetric synaptic weight update dynamics [134].

From a device-engineering perspective, the primary challenge lies in achieving linear and symmetric conductance evolution in polymer memristive synapses while ensuring robust stability, cycling endurance, and minimal device-to-device variation [134,135]. Linear synaptic weight updates are a strict requirement for achieving predictable incremental conductance evolution and stable learning behavior in ANNs, thereby eliminating the need for complex peripheral circuitry or iterative weight-correction protocols. Moreover, symmetric potentiation and depression are indispensable for faithfully implementing spike-timing-dependent plasticity and other core synaptic functionalities that govern learning and memory in neuromorphic systems [135].

To overcome these intrinsic limitations, diverse material modification strategies have been reported.

### 5.1. Material Engineering Strategies for Enhanced Linearity and Symmetry

This section focuses on material-centric engineering strategies that directly address these challenges: polymer doping to regulate ionic transport, hydrogen-bond-mediated interface engineering for interfacial stabilization, and multilayer structural design to enable distributed switching.

#### 5.1.1. Polymer Doping for Controlled Ion Migration

**Figure 8:**
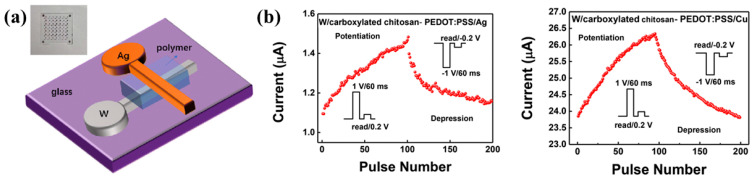
(**a**) Schematic illustration of the vertical Ag/carboxylated chitosan-PEDOT: PSS/W and Ag/carboxylated chitosan/W devices, (**b**) potentiation/depression behaviors of the Ag/carboxylated chitosan—PEDOT: PSS/W device and Cu/carboxylated chitosan—PEDOT: PSS/W device. Reuse with permission from ref. [27].

One effective strategy for achieving linear conductance modulation in polymer memristors is the rational design of composite materials that precisely control ionic transport and redox activity. For instance, a representative system employs carboxylated chitosan as an ion-conducting polymer host, lightly doped with PEDOT: PSS as a conductive modifier. In this architecture, the chitosan matrix facilitates metal-ion migration, while the incorporated PEDOT: PSS plays a dual role by enhancing ionic conductivity and stabilizing ion-induced redox processes—Figure 8a. This synergistic interaction suppresses stochastic filament growth and promotes the formation of spatially uniform conductive pathways. As a result, the device exhibits highly linear and reproducible conductance evolution, supporting over 100 stable and non-volatile conductance states within a low operating voltage window of approximately 1 V—Figure 8b [27].

#### 5.1.2. Interface Engineering and Structural Design

**Figure 9:**
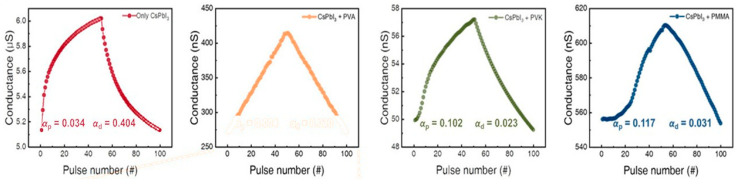
Potentiation/depression behaviors of device with different polymer interface engineering. Reuse with permission from ref. [25].

In addition to bulk material design, precise control over interfacial properties plays a decisive role in achieving linear and symmetric switching behavior in memristive devices. Interface engineering based on hydrogen–bond interactions has emerged as an effective approach, particularly in architectures utilizing polyvinyl alcohol (PVA). Owing to its dense hydrogen-bonding network, PVA can form mechanically and electronically stabilized interfaces, such as those with perovskite active layers, which effectively constrain ionic dynamics at the interface. This interfacial confinement suppresses stochastic ion migration and interfacial instability, thereby enabling highly linear and symmetric optoelectronic conductance modulation during repeated switching cycles Figure 9 [25].

#### 5.1.3. Advanced Polymer Systems for High Uniformity and Yield

For practical large-scale integration, linear conductance modulation must be accompanied by high fabrication yield and minimal device-to-device variability. Recent advances in polymer molecular engineering address these requirements by promoting long-range structural order within the active layer. Bin et. present a two–dimensional conjugated polymer PBDTT-BQTPA, developed through a planar π-conjugation design strategy. This molecular architecture enhances backbone planarity and intermolecular packing, leading to highly crystalline and morphologically uniform thin films. Such microstructural homogeneity effectively suppresses defect-induced electric-field localization, including those arising from grain boundaries or voids, which are commonly responsible for stochastic RS. As a result, memristors based on this polymer operate via bulk-dominated switching mechanisms and deliver a combination of desirable performance metrics, including high fabrication yield (~90%), nanosecond-scale switching speed, low operating voltages, long-term retention, and outstanding endurance. Importantly, the narrow distribution of key electrical parameters across device—scalable neuromorphic hardware implementations [29].

#### 5.1.4. Thermal Activation and Temperature-Dependent Mechanisms

**Figure 10:**
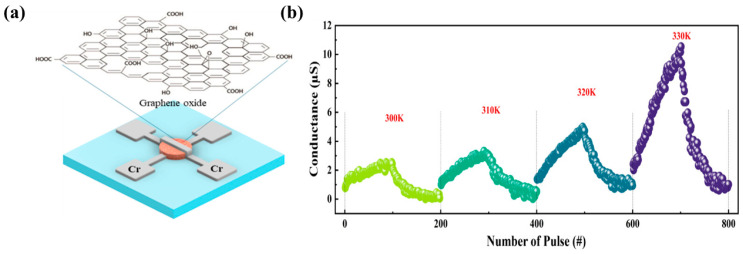
(**a**) Schematic illustration of device structure, (**b**) potentiation/depression behaviors of the device with different temperature. Reuse with permission with ref. [26].

Thermal modulation can be intentionally exploited as an effective tuning parameter to improve synaptic linearity in memristive systems. In composite-based devices such as graphene oxide–poly (vinyl alcohol) (GO–PVA), systematic temperature-dependent investigations reveal a clear evolution in charge transport behavior—Figure 10a. At lower temperatures, electrical conduction is primarily governed by space-charge-limited current (SCLC) and variable-range hopping (VRH), reflecting localized carrier transport and trap-dominated processes. As the temperature increases, enhanced thermal activation and partial reduction of graphene oxide progressively favor Ohmic and Schottky conduction pathways. This thermally induced transition in transport mechanisms directly contributes to more uniform and controllable conductance modulation, leading to markedly improved linearity and precision in long-term potentiation and depression (LTP/LTD) behavior—Figure 10b [26].

The strategies discussed above demonstrate that material engineering, interface optimization, and device-structure control can significantly improve conductance linearity and symmetry. These improvements directly influence the ability of polymer memristors to emulate biological synaptic functions. Therefore, the following section examines the key artificial synaptic characteristics enabled by polymer-based memristive devices.

## 6. Artificial Synapse

### 6.1. Key Features

A biological synapse consists of three key elements: the presynaptic neuron, the synaptic cleft, and the postsynaptic neuron. In memristive devices, these functional components are analogously represented by the BE, top electrode and the RS active layer, respectively. Information processing and storage in neural systems occur through the regulation of synaptic strength, commonly referred to as the synaptic weight. In artificial synapses based on memristors, variations in synaptic weight are emulated through controllable conductance changes, which arise from ion migration and redistribution within the active layer under external electrical stimuli. For memristors to function as artificial synapses, they must support analog switching characteristics, enabling gradual and controllable changes in conductance rather than abrupt binary transitions [136]. RS can occur either as digital switching, involving sharp transitions between high- and low-resistance states, or as analog switching, in which the device resistance is tuned progressively, a feature that is essential for emulating synaptic weight updates. Multiple conductance states arise from continuously tunable RS. The RS behavior of memristive devices is controlled by the metal–active layer interface. Devices with Schottky contacts at both electrodes exhibit digital switching, while analog switching occurs when one interface is Schottky and the other is Ohmic [137]. Resistive memory and artificial synapses operate through comparable atomic-scale mechanisms but require distinct evaluation approaches.

### 6.2. Paired-Pulse Facilitation (PPF)

**Figure 11:**
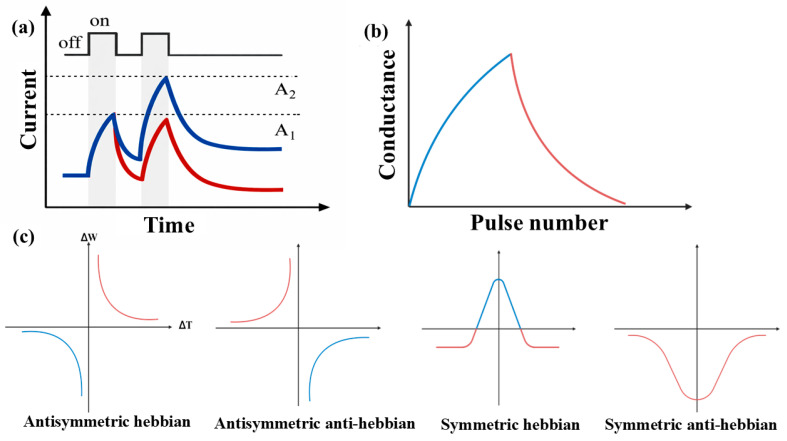
Schematic diagram of synaptic plasticity. (**a**) PPF, (**b**) LTP and LTD, (**c**) STDP.

PPF is a type of short-term synaptic plasticity in which two presynaptic voltage pulses are applied in rapid succession. This results in an enhanced excitatory postsynaptic current, where the response to the second pulse (A_2_) exceeds that of the first pulse (A). The PPF index, expressed as a percentage, is calculated from the ratio of two successive excitatory postsynaptic current (EPSC) responses, as defined in Equation (1) and as shown in Figure 11a. The PPF behavior exhibits an exponential dependence on the inter-stimulus interval (Δ*t*), with the facilitation strength increasing as Δ*t* decreases.

(3) $$PPF=\left(A_{2}/A_{1}\right)\times 100$$

### 6.3. Dynamic Range

The dynamic range describes the ratio of the maximum conductance (*G_max_*) to the minimum conductance (*G_min_*). This parameter directly influences the weight-mapping capability of artificial synaptic devices and is defined as follows

Dynamic range = *G_max_*/*G_min_* (4)

### 6.4. Number of States

The number of states represents the count of stable conductance levels that a device can achieve within its dynamic range, usually defined by the number of applied programming pulses. It is typically determined by the number of applied programming pulses.

### 6.5. Short-Term Potentiation/Depression (STP/STD)

STP/STD describes a volatile synaptic response in which the conductance change induced by electrical stimulation decays rapidly when the applied bias is below a critical threshold. After the stimulus is removed, the synaptic weight relaxes back to its initial state, analogous to biological short-term memory.

### 6.6. Long-Term Potentiation/Depression (LTP/LTD)

LTP/LTD are analogous to long-term memory. When electrical stimulation exceeds a threshold, the device’s conductance remains altered even after the external stimulus is removed, thereby exhibiting nonvolatile behavior as shown in Figure 11b.

### 6.7. Spike Rate, Width, and Voltage—Dependent Plasticity

Spike-rate, pulse–width, and voltage–dependent plasticity describes how the PPF index varies with the properties of the applied electrical pulses. Specifically, it depends on the frequency, duration, and amplitude of these pulses. Plasticity denotes the capability of a synapse, or the conductance state of a memristor, to be altered by electrical stimulation [138].

### 6.8. Spike Timing-Dependent Plasticity (STDP)

STDP describes a learning rule in which the relative timing (Δ*t*) between pre- and postsynaptic spikes defines both the magnitude and direction of synaptic weight change. When the presynaptic spike arrives before the postsynaptic spike (Δ*t* > 0), potentiation is induced, whereas a reversed sequence (Δ*t* < 0) results in depression. The magnitude of the weight update increases as the absolute time difference |Δ*t*| decreases, as shown in Figure 11c [139].

### 6.9. Linearity

Linearity quantitatively describes the uniformity of conductance (G) modulation in response to successive programming pulses. In an ideal artificial synapse, each pulse induces an identical synaptic weight update, which is critical for achieving high computational accuracy in neuromorphic systems. However, experimentally measured conductance evolution often deviates from this ideal behavior. To evaluate this nonlinearity, a linearity factor (*ν*) is extracted by fitting the conductance–pulse relationship during potentiation and depression using Equations (4) and (5).

(5) $$G_{P}=G_{min}+B\left(1-e^{-\nu N}\right)$$

(6) $$G_{D}=G_{max}-B\left(1-e^{-\nu \left(N-N_{max}\right)}\right)$$

(7) $$B=\left(G_{max}-G_{min}\right)/\left(1-e^{-\nu N_{max}}\right)$$

*G_max_* and *G_min_* are the highest and lowest conductance levels, whereas *N* is the number of pulses during potentiation and depression. A linearity factor that is closer to 0 means that the behavior is more linear, which is good for dependable computation [140].

### 6.10. Symmetry

Symmetry indicates how uniformly the device conductance changes during potentiation and depression processes. It is typically evaluated by calculating the absolute difference between the nonlinearity values associated with conductance increase and decrease

(8) $$Asymmetry=\left|\left|\alpha_{p}\right|-\left|\alpha_{d}\right|\right|$$

### 6.11. Endurance

Endurance can be divided into two forms. The first is two–state endurance, which describes the device’s ability to maintain stable switching characteristics over repeated SET/RESET operations. The second parameter is synaptic endurance, which measures the stability of LTP and LTD operations and is typically expressed by the cycles [141].

### 6.12. Retention

Retention refers to the maximum period during which a synaptic device can preserve a particular conductance state, a property that is crucial for reliable inference operations. Random fluctuations in conductance may degrade computational accuracy [142].

### 6.13. Variation

Variation is typically categorized into two primary forms: device–to–device variation and cycle–to–cycle variation. For large-scale neuromorphic hardware, controlling these variations is essential, as lower variability reduces stochastic effects, improves operational stability and reliability, and supports consistent and accurate neural computation [143].

### 6.14. Yield

Yield is defined as the proportion of fabricated devices that can be consistently programmed into multiple resistance states. This metric is particularly important for large-scale integration, as high yield is essential for maintaining stable and reliable computational performance [144].

The inference stage primarily involves data transfer between arrays, including the conversion between voltage and current signals and the quantization of analog output signals [145]. Accordingly, stricter control of dynamic range, yield, and endurance is necessary because each device must maintain stable conductance levels. The fidelity with which synaptic behavior is reproduced therefore has a direct influence on inference accuracy [146].

During the learning process, determining the optimal synaptic weights requires precise modulation of the device conductance states. Consequently, parameters associated with long-term plasticity, such as number of conductance levels, dynamic range, symmetry and the linearity, are key performance considerations [147]. In general, lower nonlinearity indicates that the conductance change produced by successive pulses is largely independent of the current conductance level, enabling more efficient and accurate updates of synaptic weights. Improved symmetry enables more precise adjustment of synaptic weights by avoiding abrupt conductance variations. In contrast, poor linearity and symmetry in conductance modulation tend to concentrate conductance states near the limits of $G_{max}$ and $G_{min}$, making the intermediate conductance levels harder to achieve. Such non-ideal weight modulation can hinder the learning process, as it reduces recognition accuracy and slows convergence during training, ultimately increasing both time and energy consumption. A limited conductance dynamic range causes intermediate conductance levels to become closely spaced, which increases errors during read and write operations. In general, increasing the dynamic range initially improves recognition accuracy, but this improvement eventually reaches a saturation point. A narrow dynamic range can also reduce the efficiency of learning convergence [145]. Devices that exhibit a large dynamic range (>10) while maintaining low conductance levels (typically 1 μS to 10 nS) are generally preferred [146]. When the device exhibits good linearity and a sufficient on/off ratio, increasing the number of accessible conductance states generally leads to improved recognition accuracy [148]. The number of available conductance states plays a complicated role in determining the convergence behavior of neuromorphic devices. In highly linear devices, increasing the number of conductance levels typically reduces the magnitude of conductance variation during each weight update, which can slow the convergence process during training. In contrast, devices exhibiting lower linearity often produce larger conductance changes per update step, leading to faster convergence. Consequently, the optimization of memristive synaptic devices requires a careful balance among different performance parameters in order to achieve an appropriate compromise between computational accuracy and convergence speed [145].

Table 3 compares polymer memristor-based artificial synapses with chalcogenide, nitride, and oxide-based synaptic devices in terms of device structure, potentiation/depression nonlinearity, and energy consumption. Polymer-based synapses show relatively low nonlinearity and low energy consumption, indicating their strong potential for linear and symmetric conductance modulation in neuromorphic computing applications.

The synaptic parameters discussed in this section, including plasticity, dynamic range, linearity, symmetry, endurance, retention, variation, and yield, collectively determine the suitability of polymer memristors for neuromorphic computing. To further clarify their relative advantages and limitations, the next section compares the performance of representative polymer-based synaptic memristors with other reported memristive systems.

## 7. Performance Comparison

A defining characteristic of such computing devices is their ability to learn, enabled by analog neuromorphic switching. All devices listed in Table 4 demonstrate fundamental synaptic plasticity behaviors, including LTD, LTP, and energy consumption. In addition, low energy consumption remains a highly desirable characteristic. The required properties vary depending on the specific application and product requirements. The performance of an artificial synapse is generally evaluated using several key parameters: (i) the voltages essential for LTP and LTD are particularly important, as they define the operating range for analog switching; (ii) the spike duration, which determines the switching speed; (iii) the device thickness; and (iv) the energy consumption per spike, which influences its mechanical flexibility. As shown in Table 4, most binary metal oxide–based devices operate with relatively low LTP and LTD voltages and energy consumption in the range of 1–25 pJ per spike. Moreover, many devices achieve switching times far below biological timescales, operating in the microsecond-to-nanosecond range [1]. It is worth noting that the device reported by Hsieh et al. exhibited an exceptionally large weight change, approximately 30 times greater than the average observed in other synaptic devices. Their energy consumption is low, with most devices operating at the picojoule (pJ) level per spike [159]. The superior performance can be attributed to the high density of oxygen vacancies in the metal oxide, which promotes the CFs formation. Most studies on perovskite-based devices do not report the current–voltage pulse relationship, leaving their energy consumption unclear. Very few perovskite–based devices with calculable energy consumption have been reported; Zhao et al. reported that the device required 677 nJ per spike, indicating a high energy dissipation. Many devices exhibit low LTP and LTD voltages, an advantage for synaptic applications [160]. Their switching speeds are fairly uniform, typically falling between 0.25 and 2 ms. Although not exceptionally fast, this range is generally considered acceptable. Organic–inorganic hybrid perovskites (OHPs) exhibit the lowest average energy consumption among all material systems, with most devices operating at the femtojoule (fJ) per spike level. They also demonstrate very low LTP and LTD voltages, which can be attributed to enhanced ion migration facilitated by the higher defect density introduced by the organic component. However, despite their superior energy efficiency and low operating voltages, most devices still suffer from relatively slow switching speeds. In contrast to metal oxide–based devices, which exhibit relatively consistent energy consumption, polymer- and organic-based devices show a wide variation in both energy consumption and operating voltage, ranging from high to low values. Therefore, it is challenging to characterize their energy behavior using a single standard. Further improvements are needed in their switching speeds, which typically range from 1 to 100 ms. Their primary advantages lie in their mechanical flexibility, thermal stability, and environmentally friendly processing.

Quantitative performance comparison with inorganic oxide synapses is necessary to evaluate the relative performance of polymer-based memristors for neuromorphic computing. As summarized in this review, polymer synaptic devices can achieve low energy consumption in the fJ-to-pJ range, with PET/ITO/PEDOT: PSS/ITO and Au/DPDA/ITO devices exhibiting 252 fJ and 20.9 fJ per synaptic event, respectively. In contrast, a Ti/HfO_2_/HfO_2−x_/Pt oxide synapse shows an energy consumption of 137 pJ per event, set/reset voltage coefficients of variation of 11.8% and 13.22%, retention above 10^4^ s, and endurance over 4000 pulse cycles [12]. Moreover, Ta_2_O_5−x_/TaO_2−x_ bilayer oxide memristors exhibit fast switching of approximately 10 ns and endurance exceeding 10^12^ cycles, indicating their strong reliability for high–density neuromorphic integration [122]. These comparisons show that polymer–based memristors offer distinct advantages in flexibility, molecular tunability, low-temperature processing, solution processability, and large-area fabrication, while HfO_x_/TaO_x_ oxide memristors remain important benchmark systems for dense and highly reliable inorganic neuromorphic arrays.

Comparison with perovskite- and 2D-material-based memristors. To provide a broader perspective on emerging memristive materials, the performance of polymer-based artificial synapses can be compared with those of 2D-material-based devices and perovskite devices. 2D materials, including transition-metal dichalcogenides and borophene/graphene-based hybrids, provide atomically thin channels, high surface-to-volume ratios, strong electrostatic control, and compatibility with transparent or flexible device structures [77,78,153]. These characteristics are beneficial for scaling and low-power operation; nevertheless, transfer-induced defects, grain boundaries, contact resistance, and nonuniform defect or filament formation can increase cycle–to–cycle and device-to-device variability. For example, the Ag/WTe2/Pt synapse listed in Table 3 shows relatively high potentiation/depression nonlinearity values of 2.47/1.76, whereas optimized polymer–perovskite hybrid synapses exhibit much lower nonlinearity values of 0.004/0.020 and 0.013/0.006, respectively [25,151,153]. Therefore, excellent intrinsic electrical properties do not always translate directly into ideal analog weight-update behavior. Perovskite systems are attractive because their mixed ionic–electronic transport and soft crystal lattice enable low-voltage conductance modulation, as summarized in Table 4. It is indicated that representative perovskite and halide–perovskite synapses operate at LTP/LTD pulse voltages of 0.2–2.5 V, with reported energy consumption ranging from the attojoule/femtojoule regime to much higher values when leakage current or current compliance is not well controlled [160,161,164,165]. However, ion migration, phase instability, moisture/thermal sensitivity, and potential toxicity in Pb-containing compositions can limit long-term reliability and practical integration. Compared with these emerging materials, polymer-based memristors generally offer advantages such as low–temperature fabrication, mechanical compliance, chemical tunability, and large-area processability. Their main limitations are slower switching, wider energy dispersion, and sensitivity to morphology, electrode selection, and environmental conditions. Thus, polymer memristors are particularly competitive for flexible, wearable, and large-area neuromorphic systems, whereas perovskite and 2D–material memristors are attractive for ultralow-power and highly scaled devices, provided that stability, variability, and CMOS–compatible processing can be further improved.

The comparative analysis shows that polymer-based memristors offer attractive advantages in terms of flexibility, tunability, low-temperature processing, and synaptic functionality. However, their ultimate value is determined by their ability to support practical neuromorphic computing tasks. Therefore, the following section discusses polymer-based systems for neuromorphic computing, with emphasis on pattern recognition and system-level performance.

## 8. Polymer-Based Systems for Neuromorphic Computing

In addition to optimizing artificial synapses, researchers are also working to integrate them with external devices into a single system. Among the various applications of artificial synaptic devices, neuromorphic computing tasks, particularly image recognition, have attracted significant research attention.

In conventional computing, images are commonly represented as two-dimensional pixel arrays. Each pixel is encoded using 24 bits, consisting of three 8-bit values corresponding to the blue, green and red color channels. It supports up to 16, 777, 216 colors. Each pixel’s color is specified by three values ranging from 0 to 255, which indicate the intensities of red, green, and blue. These three values are then converted into binary form. Thus, a single pixel requires 24 bits of storage. For example, the color violet can be represented by the binary values “11101110, 10000010, 11101110 [169,170]. Multi-level memristors enable multiple bits to be stored in a single cell. For example, a memristor developed by the University of Southampton in 2017 demonstrated a record 92 distinct states, equivalent to 6.5 bits per cell [171]. The mainstream ANN approach to computational tasks employs an architecture with three types of layers: input, hidden, and output [172,173]. The overall system integrates an optical neural network with a conventional neural network [174]. First, the image is captured by the device’s sensor, triggering spiking activity in the input (presynaptic) neurons via leaky integrator units, with responses modulated by the memristor weights. Next, the current passes through a comparator, with its reference level set to the threshold voltage. Once the output voltage rises above $V_{\mathrm{th}}$, the output neuron generates postsynaptic spikes that propagate through feedback pathways to modify the synaptic weights [133,175]. Then, following the STDP rule, each memristor is progressively adjusted to the conductance values required to represent the original image. This process can be summarized by the following equations:

(9) $$Y_{in}=\sum_{m=1}^{\#\;of\;X}X_{m}V_{m,in}$$

(10) $$Y_{on}=\frac{1}{1+e^{-Y_{in}}}$$

(11) $$Z_{ik}=\sum_{n=1}^{\#Y}Y_{on}W_{nk}$$

(12) $$Z_{ok}=\frac{1}{1+e^{-Z_{ik}}}$$

(13) $$E=\frac{1}{2}\sum_{k=1}^{\#Z}{\left(O_{k}-Z_{k}\right)}^{2}$$

*E*, Y and Z denote three types of neurons in the network. Specifically, they correspond to the input, hidden (middle), and output neurons, respectively. Here, $V_{mn}$ defines the weights between the input and hidden layers, whereas $W_{nk}$ defines the weights between the hidden and output layers. *Y_in_* refers to the input of the hidden layer neurons. $Y_{on}$ refers to their output, while *Z_ik_* denotes the input of the output-layer neurons. $Z_{ok}$ denotes their output, respectively. denotes the output error, while $O_{k}$ represents the correct output value.

Those studies were carried out by combining photosensitive elements with electrically stimulated synapses. Despite its advantages, this architecture has two significant limitations; the first is the need for recovery speeds and rapid response. When optical stimuli are rapidly removed, the system may fail to process the optical information effectively. Secondly, similar to the von Neumann architecture, the separation of processing units leads to increased energy loss during data transfer. To address these limitations, researchers have developed optoelectronic synapses, in which optical stimuli can be processed directly within an integrated visual recognition unit.

**Figure 12:**
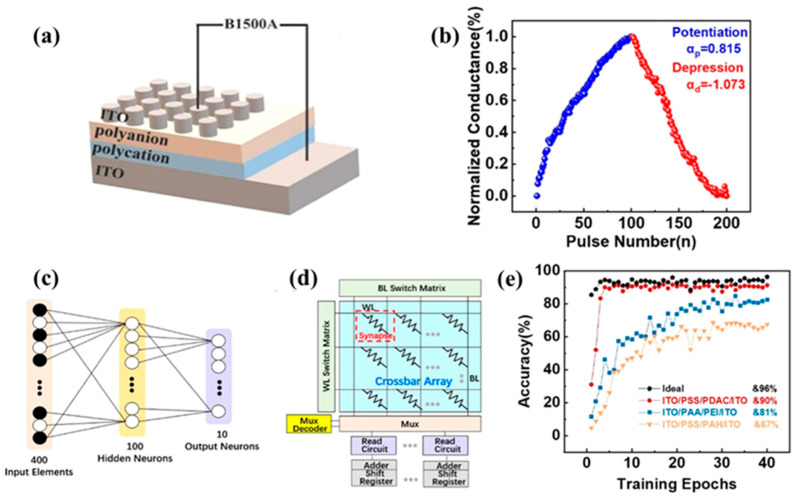
(**a**) Schematic illustration of device structure. (**b**) Potentiation/depression behaviors ITO/PSS/PDAC/ITO device. (**c**) Schematic showing the fully connected network. (**d**) Circuit structure. (**e**) Identification accuracy. Reuse with permission with from ref. [31].

Through the engineering of a nanoscale polyelectrolyte bilayer interface that regulates ion and counterion migration, a PSS/PDAC–based memristive synaptic device was developed, demonstrating stable analog RS and reliable synaptic weight modulation. The device employed an ITO/PSS/PDAC/ITO architecture, where poly (styrene sulfonate) (PSS) and poly (diallyl dimethylammonium chloride) (PDAC) formed an ultrathin active bilayer with a total thickness of approximately ~15 nm, enabling efficient ion redistribution and modulation of the interfacial potential drop under electrical stimulation (Figure 12a). The rigid aromatic structure of PSS and the sterically constrained PDAC chains suppress excessive polymer migration, resulting in a gradual evolution of the internal electric field and thus enabling highly controllable conductance modulation. The PSS/PDAC memristive synapse exhibited stable multilevel conductance states under repeated voltage pulse stimulation, showing low nonlinearity values for synaptic weight updates with potentiation nonlinearity of 0.815 and depression nonlinearity of −1.073, indicating relatively symmetric conductance modulation during learning operations (Figure 12b). Compared with the control PAA/PEI device (αp = 2.259, αd = −5.303), the optimized PSS/PDAC device substantially reduces these values to αp = 0.815 and αd = −1.073, directly confirming the improvement in both linearity and symmetry.

The experimentally measured synaptic conductance states were directly mapped as synaptic weights in a fully connected artificial neural network. The network architecture comprised 400 input neurons, 100 hidden neurons, and 10 output neurons. The network was subsequently trained on the MNIST handwritten digit dataset (Figure 12c). The corresponding circuit architecture is presented in Figure 12d, and the fully connected network was trained using 60,000 images from the MNIST dataset. The hardware-informed neural network exhibited a classification accuracy of approximately 90%. This result indicates that the device characteristics exhibit sufficient linearity and stability to support neuromorphic learning tasks, as illustrated in Figure 12e. Furthermore, the device exhibited biologically relevant synaptic plasticity behaviors, including transitions between short-term plasticity (STP) and LTP as well as short-term depression (STD) and LTD when the pulse amplitude was varied at a fixed pulse width of 100 ms, confirming the capability of the PSS/PDAC bilayer system to emulate fundamental synaptic learning processes. These results demonstrate that controlled polyelectrolyte–chain migration and interfacial electrostatic modulation provide an effective strategy for developing low–power, highly linear polymer memristive synapses suitable for neuromorphic computing applications [31].

Through the regulation of metal-ion migration and conductive filament formation in an organic polymer dielectric matrix, a polymer–based conductive–bridging memristive synaptic device was developed, demonstrating high linearity and multilevel conductance modulation suitable for neuromorphic computing applications. Carboxylated chitosan was employed as the dielectric switching layer and was doped with a small amount of the conductive polymer PEDOT: PSS, resulting in enhanced ionic conductivity and enabling precise modulation of the redox dynamics of metal ions within the device. The incorporation of PEDOT: PSS rendered the polymer film fully amorphous, creating abundant ion transport pathways. Consequently, the applied electric field effectively induces ion transport, resulting in uniform CF formation throughout the dielectric layer. Consequently, the operating voltage required for the resistance switching process was markedly reduced from >10 V in pristine chitosan devices to approximately 1 V, substantially lowering the energy consumption of the synaptic device.

The PEDOT: The PSS–modified device exhibited gradual conductance modulation and stable multilevel switching behavior owing to the formation of uniformly distributed CFs instead of randomly generated dendritic structures. The conductive polymer also acted as an intermediate electron reservoir that assisted the reduction of migrating metal ions, thereby regulating filament growth and improving switching uniformity. Furthermore, when the active electrode was replaced with Cu to control ionic activity, the resulting Cu/carboxylated chitosan-PEDOT: PSS/W memristor displayed highly linear potentiation and depression behavior under consecutive electrical pulse stimulation, enabling symmetric and controllable synaptic weight updates.

To evaluate the neuromorphic computing capability of the device, the experimentally obtained conductance states were mapped as synaptic weights in artificial neural network simulations. A backpropagation-trained neural network using handwritten digit datasets demonstrated recognition accuracies of approximately 90% for 8 × 8 images and 94% for 28 × 28 images, while document classification tasks achieved an accuracy of 86% after training iterations. These results highlight the strong potential of polymer-based conductive-bridging memristors with regulated ion transport dynamics for high-precision neuromorphic computing systems and artificial synaptic networks [27].

**Figure 13:**
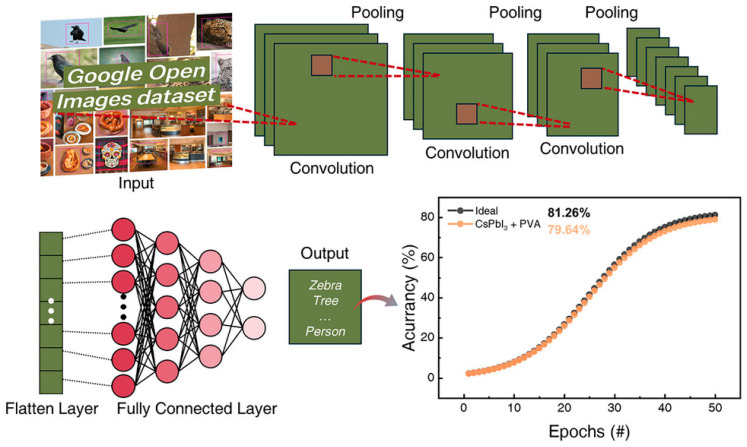
Classification accuracy. Reuse with permission from ref. [23].

A CsPbI_3_-based synaptic device was developed by engineering a polymer–perovskite hybrid interface. This interface modulates ionic migration dynamics through hydrogen-bond interactions. As a result, the device exhibited improved operational stability and reliable analog switching behavior. PVA was incorporated into the perovskite framework, where it formed strong hydrogen-bond interactions with surface iodide ions. The hydrogen-bond-mediated interface facilitates an ordered structural arrangement and vertically oriented crystallization, directing iodide ion migration along a vertical pathway and thereby enabling stable and symmetric conductance modulation. As illustrated in Figure 9, the PVA–perovskite synaptic device displays distinct multilevel conductance states under hybrid photoelectrical stimulation, exhibiting symmetric weight modulation (0.016) and improved linearity (0.004/0.020). Compared with the pristine CsPbI_3_ device before optimization, which indicates αp = 0.034, αd = 0.404, and a symmetry deviation of 0.370, it can confirm that PVA interface engineering substantially improved both linearity and symmetry. Subsequently, a two–layer artificial neural network was constructed for handwritten digit recognition on the MNIST dataset. The experimentally measured conductance states of the memristive synapse were directly assigned as synaptic weights. The model achieved a classification accuracy of 93.8%, approaching the theoretical performance limit. In addition, to evaluate the device capability for more complex large-scale image recognition tasks, an improved convolutional neural network was trained using the Google Open Images Dataset (Figure 13), resulting in a final recognition accuracy of 79.64% [25].

Polymer/biopolymer-based memristive synaptic devices are increasingly explored for neuromorphic computing because their analog, multilevel conductance modulation can emulate synaptic weight updates required for learning and pattern recognition. In this work, the AgNO_3_–doped ι–carrageenan synaptic memristor exhibited improved linearity in potentiation/depression behavior, which is critical because synaptic nonidealities (notably weight-update nonlinearity) degrade training reliability and inference accuracy in neuromorphic systems. To assess its practical relevance, handwritten digit recognition was performed on 28 × 28-pixel MNIST images using a three—layer neural architecture consisting of 784 input units, 300 hidden units, and 10 output units. The model was implemented within a crossbar–based computing framework. Rather than assuming ideal switching, they adjusted the nonlinearity (NL) parameters of the weight-update model using fitted experimental results and performed supervised learning on a crossbar simulation platform (Cross-Sim). With AgNO_3_ doping, the simulated recognition accuracy improved markedly to 93.8%, approaching the ideal linear-synapse baseline of 95.7% (and far exceeding the undoped case at 56.3%), highlighting the practical value of improving polymer memristor linearity for neuromorphic pattern-recognition tasks [176].

Polymer-based memristive artificial synapses are increasingly being investigated for neuromorphic computing because their gradual, analog, and multilevel conductance modulation can emulate the weight–update characteristics of biological synapses required for learning and pattern-recognition tasks. In this work, the LiClO_4_–doped PEDOT: PSS artificial synaptic memristor exhibited significantly improved linearity in potentiation/depression behavior, which is especially important because synaptic nonidealities, particularly nonlinear weight–update characteristics, directly limit the training efficiency and recognition performance of neuromorphic systems. To demonstrate application-level relevance, the authors implemented a face-recognition task using a three-layer artificial neural network and evaluated the synaptic characteristics of the device under both identical and incrementally modulated pulse schemes. Rather than assuming an ideal synaptic response, they extracted the conductance-update behavior from experimental device measurements and incorporated the corresponding nonlinearity factors into the neural-network simulation. Under identical pulse conditions, the device showed relatively poor linearity, with nonlinearity values of approximately 6.5 for potentiation and 6.8 for depression; however, by employing an incremental pulse-amplitude scheme from 0.8 to 2.0 V in 50 mV steps, the linearity improved substantially to αp = 1.5 and αd = 0.4. As a result, the simulated face–recognition accuracy was increased to 96%, compared with 92% for the nonlinear update case, highlighting the practical significance of improving conductance linearity in polymer–based artificial synapses for reliable neuromorphic computing applications [177].

**Figure 14:**
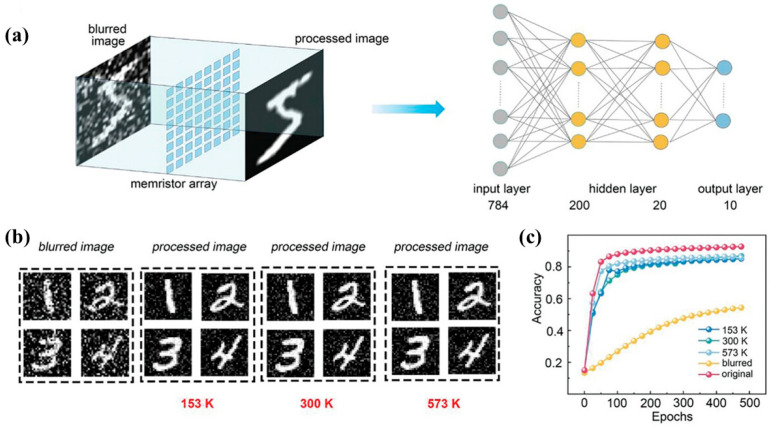
Simulated blurred-image recognition in a neuromorphic system employing PTBZ-DPP–based memristors. (**a**) Schematic of the neuromorphic architecture, where a memristor array performs image deblurring and interfaces with an artificial neural network for subsequent recognition. (**b**) Representative examples of blurred input images (**left**) and the corresponding outputs after memristor-based preprocessing. The memristor devices were thermally treated at 153, 300, and 573 K. (**c**) Comparative evaluation of recognition accuracy for original, blurred, and processed images under the same temperature conditions (153, 300, and 573 K). Reuse with permission with ref. [178].

Through the design of a PTBZ–DPP polymer memristor with intramolecular charge-transfer characteristics that enable reliable analog switching and threshold-dependent signal regulation, a neuromorphic device was developed for pattern-recognition-oriented image processing. The polymer memristor exhibited stable multilevel conductance modulation along with synaptic plasticity behaviors, including LTP and LTD. Its threshold-switching behavior further enabled selective suppression of weak background signals and amplification of feature-relevant inputs; as illustrated in Figure 14a, this capability was utilized in a memristor-array-assisted blurred image recognition system, where higher-intensity pixels were encoded using 3 V pulses and background noise with 0.5 V pulses, followed by repeated stimulation to enhance signal–to–noise contrast. Subsequently, handwritten digit recognition was performed using a four–layer artificial neural network that processed 28 × 28-pixel images. The network consisted of 784 input nodes, two hidden layers with 200 and 20 nodes, respectively, and 10 output nodes.

As illustrated in Figure 14b, the digits “1,” “2,” “3,” and “4” become significantly more distinct after denoising by the memristor array, with improved edge definition observed for devices thermal treatments at 153, 300, and 573 K. After memristor-based preprocessing, the recognition accuracy of blurred images improved markedly from 54.37% to above 85% after 500 epochs, approaching the performance obtained for the original clear images (92.78%), while maintaining robust operation over a broad temperature range as shown in Figure 14c. In addition, to evaluate the capability of the device in more complex dynamic pattern–recognition tasks, a memristor-assisted convolutional neural network (CNN) was further developed for motion–deblurred license plate recognition, in which the experimentally derived LTP/LTD characteristics were used to update synaptic weights and optimize blur–kernel estimation. This approach enabled restoration of blurred vehicle images, making previously unreadable license plate information distinguishable. These results highlight the practical significance of polymer memristors as multifunctional neuromorphic hardware for pattern recognition, denoising, and deblurring applications [179].

Table 5 summarizes representative polymer memristor devices for pattern and image recognition applications. The comparison clearly shows that lower potentiation and depression nonlinearity is closely related to improved recognition accuracy. These results highlight the importance of linear and symmetric conductance modulation in polymer-based synaptic memristors for reliable neuromorphic computing.

The system-level studies discussed above demonstrate that improved conductance linearity, multilevel switching, and device reliability can directly enhance neuromorphic learning and pattern-recognition accuracy. These results confirm the potential of polymer-based artificial synapses as functional building blocks for brain-inspired computing. The following section summarizes the main findings of this review and highlights the overall significance of polymer memristors for accurate and reliable neuromorphic systems.

## 9. Conclusions

Polymer-based memristive artificial synapses have emerged as a highly promising platform for next-generation neuromorphic computing, owing to their tunable physicochemical properties, intrinsic ionic–electronic coupling, mechanical flexibility, and compatibility with low-temperature, large-area fabrication. This review has established a clear connection between fundamental switching mechanisms, such as ionic migration, charge transfer, charge trapping/detrapping, conformational dynamics, conductive filament formation, redox reactions, and device performance, emphasizing their critical role in achieving stable and analog synaptic behavior. Through systematic classification and analysis of device architectures, it is evident that material design and interface engineering are key to improving conductance modulation characteristics.

In particular, achieving linear and symmetric weight updates remains central to enhancing learning accuracy in neuromorphic systems. Recent advances in controlled doping, interface optimization, and structural design have significantly improved linearity, reduced variability, and enabled reliable multilevel states. These improvements directly translate into enhanced synaptic functionalities, including plasticity, dynamic range, endurance, and energy efficiency, thereby enabling more accurate pattern recognition and neuromorphic computing tasks.

Despite this progress, challenges such as device variability, limited long-term stability, and large-scale integration persist. Addressing these issues will require mechanism-driven material design, improved control over ion dynamics, and the development of scalable, CMOS–compatible fabrication strategies. Overall, polymer-based artificial synapses provide a viable platform for achieving accurate, reliable, and energy-efficient neuromorphic systems. Continued interdisciplinary research is expected to drive their advancement from fundamental studies toward practical intelligent applications.

Consequently, scalability should be interpreted as an array-level challenge rather than a direct consequence of promising single device behavior. For practical crossbar implementation, polymer synaptic memristors must suppress sneak—path currents through selector integrated architectures, such as one-selector-one-memristor or one-transistor-one-memristor cells, or through intrinsic self-rectifying behavior. Therefore, reliable large-scale deployment will require combined verification of P/D linearity, selector compatibility, half-select disturbance immunity, read-margin stability, and array-level learning accuracy.

While the preceding sections summarize the progress and promise of polymer-based artificial synaptic memristors, several obstacles still limit their practical deployment. Issues such as device variability, long term stability, reproducibility, large–area integration, and environmental sustainability must be addressed before these devices can be widely implemented. Therefore, the next section discusses the major challenges and corresponding strategies for advancing polymer-based neuromorphic technologies.

## 10. Challenges and Corresponding Strategies

Despite significant progress in the research and deployment of polymer-based memristors and artificial synapses, several critical challenges persist for neuromorphic computing. Key issues include limited material stability and long-term reliability, poor reproducibility and cost-effectiveness of fabrication processes, and challenges associated with device integration and packaging. More importantly, for accurate neuromorphic computing, the central unresolved challenge is not simply device integration or general manufacturability, but the ability of the memristive device to obtain repeatable, linear, and symmetric potentiation/depression (P/D) behavior over many conductance states and operating cycles. Nonlinear conductance updates usually arise from abrupt filament formation, saturation of redox centers, nonuniform trap filling, or excessive local electric fields, whereas P/D asymmetry is commonly caused by imbalanced ion migration, unequal carrier-injection barriers, and different SET/RESET kinetics. These effects distort the intended synaptic weight update and directly reduce pattern-recognition accuracy during hardware–based learning. Therefore, in this section, we discuss the major challenges facing polymer-based memristors, with particular emphasis on the material, interfacial, and device-engineering factors that determine analog switching linearity, symmetry, stability, and scalability.

A first challenge is the mechanistic control of gradual conductance evolution. In polymer–metal-ion and filamentary systems, high ion mobility can provide low-voltage switching but may also generate dendritic or localized conductive paths that produce abrupt SET events. Corresponding strategies for these issues include reducing the free-ion concentration, introducing ion-coordination sites, using cross-linked or block-copolymer matrices to confine ion motion, and incorporating interfacial layers that suppress uncontrolled metal injection. The goal is to transform stochastic filament growth into spatially distributed, reversible, and pulse–rate–controlled conductance modulation.

A second challenge is the imbalance between potentiation and depression. Even when many intermediate conductance states are available, different energy barriers for carrier injection and extraction can lead to asymmetric update slopes under opposite pulse polarities. This issue should be addressed through paired electrode selection, interface dipole control, buffer layers, and polymer energy-level engineering. In addition, symmetric or deliberately balanced electrode/polymer interfaces can reduce the difference between forward and reverse switching kinetics, while optimized pulse amplitude, width, and interval can further compensate for residual asymmetry without sacrificing energy efficiency.

A third challenge is device–to–device and cycle–to–cycle variability in the linearity and symmetry metrics themselves. Variability originates from thickness fluctuations, nanoparticle aggregation, uneven trap distributions, rough interfaces, and local hot spots in large-area films. Scalable coating, printing, and patterning methods should therefore be evaluated not only by yield and ON/OFF ratio, but also by the statistical spread of P/D nonlinearity factors, asymmetry index, write noise, dynamic range, and state retention. For polymer–nanoparticle and polymer–heterojunction devices, uniform dispersion and stable interfacial coupling are especially important because aggregation or phase separation can convert analog switching into localized, nonlinear switching.

A fourth challenge is the suppression of sneak path currents in high-density crossbar arrays. Even when an individual polymer synaptic device exhibits linear and symmetric potentiation/depression, unselected or half selected cells in a passive crossbar can create parasitic current paths during read and write operations. These leakage paths can distort the measured conductance, reduce the read margin, increase power consumption, induce unintended weight updates, and ultimately degrade neuromorphic inference and training accuracy. Thus, practical scalability cannot be concluded from single-device switching characteristics alone. The performance of polymer-based synapses should be evaluated together with access devices or intrinsic rectification strategies, including one-selector-one-memristor (1S1R), one-transistor-one-memristor (1T1R), diode-coupled cells, complementary resistive switching, or self-rectifying polymer heterojunctions. In polymer systems, self-rectification may be engineered through asymmetric electrode work functions, interfacial barrier layers, ionic–blocking contacts, or donor–acceptor heterojunctions; however, these approaches must be designed so that rectification does not compromise gradual and balanced P/D conductance modulation. Future studies should therefore report selector compatibility, rectification ratio, half-select disturbance, read/write margin, wire-resistance effects, and crossbar-level simulations using experimentally measured device parameters before claiming accurate and reliable large-scale neuromorphic implementation.

Coating and casting techniques are widely employed for fabricating functional thin films in artificial synapses, owing to their simplicity and cost-effectiveness. However, achieving uniformity at scale remains a major limitation for practical applications, a challenge that becomes increasingly pronounced in multilayer integration of functional films [182,183]. Therefore, advanced patterning techniques are essential for fabricating large-area films with uniform and well-defined features, enabling controlled and oriented deposition and improving the tunability of individual device elements. In this regard, printing technologies hold significant promise for artificial neural network fabrication, owing to their precise compositional control, high-throughput processing, compatibility with diverse large-area substrates, and capability for low-cost, rapid prototyping.

Traditional polymer-based memristor for neuromorphic devices suffer from low yield and poor reproducibility, which significantly hinder their scalability for large-scale integration. Chen et al. employed advanced nanofabrication techniques to enhance the coplanarity, crystallinity, and RS stability of the 2D conjugated polymer PBDTT–BQTPA for memristor applications. This approach enabled the development of a nanoscale heterojunction neuromorphic memristor with a high device yield of up to 90% and low power consumption. Notably, the device demonstrated excellent operational stability and fast response characteristics across a wide dimensional range, from hundreds of nanometers to hundreds of micrometers.

The challenges discussed above indicate that future progress will depend on coordinated advances in materials chemistry, device engineering, scalable fabrication, integration technology, and system-level design. Building on these considerations, the following section outlines future perspectives for polymer-based memristive artificial synapses and their potential role in next-generation neuromorphic computing systems.

## 11. Future Prospectives

Polymer-based synaptic memristors are considered promising candidates for future neuromorphic computing systems because of their mechanical flexibility, chemical tunability, low-temperature processability, and compatibility with large-area fabrication. However, their future development should be guided more specifically by the central requirement of linear and symmetric conductance modulation. In artificial synapses, accurate potentiation and depression behavior is essential for reliable weight updating, stable training convergence, and high learning accuracy. Therefore, future research should focus not only on achieving resistive switching or multilevel memory characteristics, but also on developing polymer memristors that can provide gradual, reproducible, and balanced analog conductance modulation.

A major future direction is to establish clear structure mechanism performance relationships for polymer-based synaptic memristors. The polymer backbone structure, side-chain polarity, ion-binding strength, trap distribution, molecular packing, electrode work function, and interfacial energy barriers all strongly influence conductance evolution during potentiation and depression. A deeper understanding of these relationships would allow polymer materials to be selected or synthesized for controlled analog weight updates rather than for memory switching alone. Such knowledge is essential for identifying the chemical and physical origins of P/D linearity, P/D symmetry, dynamic range, write noise, and device-to-device variation.

One promising opportunity lies in the molecular design of polymers that support distributed and reversible charge modulation. Redox-active side groups, donor–acceptor backbones, zwitterionic moieties, and coordinated ion-conducting domains can be engineered to suppress abrupt filament formation while maintaining a sufficiently large conductance window. In addition, polymer heterojunctions and polymer nanocomposites should be designed with uniform interfaces and well–controlled trap landscapes. Nanoparticles, two-dimensional fillers, and interfacial layers can improve analog resolution and multilevel conductance states, but they must be incorporated carefully to avoid aggregation, localized switching hot spots, and asymmetric conductance updates.

Memristor-based neural network computing is considered a promising approach for future computing systems. A major transformation in the field of neuromorphic computing is expected in the near future. The field of neuromorphic computing is poised for a significant transformation. Neuromorphic computing, which focuses on designing hardware and software that emulate the structure and functionality of the human brain, holds substantial potential to transform artificial intelligence and digital technologies. Advances in material systems, innovative device architectures, and emerging operating mechanisms of the memristive devices are expected to further accelerate progress and enhance the capabilities of next-generation AI systems. The ultimate objective of neuromorphic device development is to realize systems that closely emulate the neural networks of biological brains. Achieving this goal would help bridge the gap between advanced non-volatile memory (NVM) technologies and their practical implementation in machine learning, thereby enabling more efficient, adaptive, and sustainable artificial intelligence systems. In recent years, the advancement and deployment of system-on-chip (SoC) solutions for accelerating neural network (NN) computing have faced considerable challenges, particularly in specialized applications such as keyword spotting, image recognition, natural language processing and wearable devices.

Successfully resolving these limitations is essential to unlock the full capabilities and benefits of neuromorphic device–integrated system-on-chip (SoC) platforms. Moreover, the convergence of these advanced technologies is expected to fundamentally transform neuromorphic computing and artificial intelligence, driving significant progress in algorithm–circuit–architecture co-design, event-driven processing, and emerging neural network structure. Consequently, further exploration of biologically inspired synaptic mechanisms and the capabilities of neuromorphic devices present vast opportunities for realizing embodied neuromorphic intelligence, enabling the implementation of complex brain-like functions in hardware systems.

Progress in neuromorphic computing relies on interdisciplinary collaboration, integrating expertise from computer science, electronics, and materials science. Each field brings indispensable expertise, encompassing novel materials development, innovative device architecture design, and the development of advanced computational algorithms. Addressing the inherent complexities of neuromorphic systems and enabling their effective translation into real-world applications requires a multidisciplinary approach.

The absence of well-defined industry standards for neuromorphic devices and materials reveals a major challenge at the present stage. Without consistent evaluation criteria, unified device architectures, and standardized material systems, it becomes difficult to ensure interoperability, maintain reliability, achieve scalable production, and fairly compare different technological approaches. The establishment of standardized frameworks is essential to enable widespread adoption, promote effective collaboration, and accelerate technological advancement of neuromorphic computing in industrial applications.

Another important future direction is the development of standardized evaluation protocols specifically for linearity and symmetry. For meaningful comparison among different polymer synaptic memristors, future studies should report potentiation/depression curves under identical pulse conditions, extracted nonlinearity parameters, asymmetry indices, device–to–device distributions, cycle–to–cycle variation, write noise, number of stable conductance states, retention of intermediate states, and learning accuracy obtained using experimentally measured update curves. Such targeted benchmarking is more directly relevant to polymer-based artificial synapses than broad discussions of general industry standards because it directly connects device physics with neuromorphic computing performance.

Device algorithm co–design is also expected to play a critical role in the future development of polymer synaptic memristors. Even carefully engineered polymer devices may still exhibit residual nonlinearity, asymmetry, drift, and stochastic fluctuation during repeated pulse operation. Therefore, pulse–engineering schemes, closed-loop write–verify methods, adaptive training algorithms, and hardware-aware neural-network models should be developed together with the material platform. The main objective is not only to reduce device imperfections, but also to quantify them and incorporate them into training rules so that array-level learning accuracy remains robust.

Scalable fabrication is another key requirement for practical polymer–based neuromorphic systems. Large-area printing, low-temperature processing, and flexible substrates are attractive for polymer electronics, but these advantages are meaningful for synaptic applications only if the resulting devices maintain narrow distributions in P/D linearity and symmetry across large arrays. Future studies should therefore combine morphology control, thickness monitoring, interface passivation, and array-level electrical mapping to ensure that the gradual conductance modulation observed in single devices can be retained in crossbar arrays and flexible neuromorphic circuits. This is particularly important because polymer morphology, phase separation, crystallinity, ionic mobility, and electrode contact quality are highly sensitive to processing conditions.

At the practical array level, future work should integrate sneak-path mitigation into the earliest stages of polymer device design. Materials that provide excellent analog synaptic modulation should also be tested under realistic crossbar-biasing schemes because selector nonlinearity, self-rectification, line resistance, and read-disturb effects can change the apparent conductance update observed by the neural-network circuit. Therefore, polymer synaptic arrays should be benchmarked using hardware-aware models that include selector behavior and parasitic currents, rather than using ideal crossbar assumptions.

**Figure 15:**
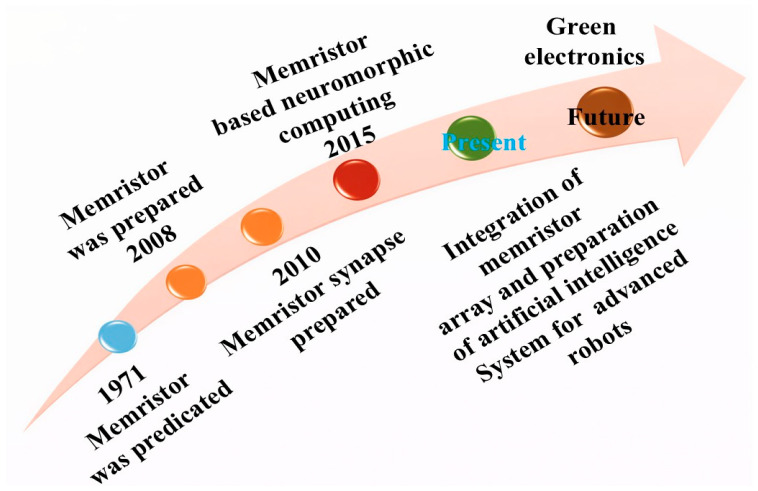
A strategic roadmap for advancing memristors and memristor-based nanoelectronic devices toward next-generation, energy-efficient AI systems.

From a broad perspective, we classify the evolution of memristor-based memory storage technologies into four distinct phases, as illustrated in Figure 15. The first phase (1971–2008) highlights the evolution of RS in single–device memristors. The second phase (2008–2010) focuses on emulating human synaptic behavior. The third phase (2008–2015) marks the emergence of high-density memristor arrays enabling artificial neuromorphic computing. Finally, the fourth phase (from 2015 to the present) ushers in a new era of green electronics, with memristor-based AI systems advancing robotic applications. In this recent phase, research has increasingly shifted toward sustainable and energy-efficient memristive platforms based on biomaterials, biopolymers, and green organic materials. These materials offer biodegradability, biocompatibility, flexibility, renewability, and low-cost processing, making them suitable for wearable, implantable, and environmentally responsible neuromorphic electronics [64]. Therefore, developing green electronics has become imperative to reduce the environmental burden caused by the alarming rise in electronic waste. In addition, green-synthesized 2D organic-polymer memristors with recyclable-electrode compatibility have shown synaptic behavior, image denoising, edge detection, and nearly 10^3^-times lower power consumption than GPU-based processing, highlighting their promise for sustainable AI hardware [184]. Therefore, green memristor-based AI hardware is expected to support compact, low-power, wearable, implantable, and environmentally responsible neuromorphic systems for future intelligent robotic applications.

Neuromorphic devices must preserve operational stability and performance integrity under varying conditions over extended periods, ensuring long–term reliability. This necessitates targeted research and development, continued progress in materials science, and rigorous testing frameworks to address system degradation and enhance overall robustness. Advancing polymer materials and neuromorphic computing further requires deep, interdisciplinary expertise in computational methods, electronic systems, and materials science.

## Figures and Tables

**Table 3:** Comparison of polymer memristor-based artificial synapses with various material type memristor-based artificial synapses.

Materials	Device Structure	Non-Linearity (Potentiation)	Non-Linearity (Depression)	Energy-Consumption	Ref.
	Ag/PMMA/CsPbI_3_ + PVA/FTO	0.004	0.020	12.5 pJ	[25]
	Au/PBDTT-BQTPA/ITO	-	-	1 pJ	[29]
	PET/ITO/PEDOT: PSS/ITO	3.5	−4.1	252 fJ	[149]
	Au/DPDA/ITO	0.22	0.33	20.9 fJ	[150]
	Ag/(PDA)(FA)6Pb7I22/PEDOT: PSS/ITO	0.013	0.006	13 pJ	[151]
Chalcogenide	Ti/GeTeO_x_/Ti–Ag	0.16	0.72	-	[152]
	Ag/WTe_2_/Pt	2.47	1.76	-	[153]
Nitride	Ag/AlN/TiN	0.36	0.42	16.7 μJ	[154]
	TiN/AlN/Cu/AlN/Pt	2.39	2.19	-	[155]
Oxide-based	Ag/SZO/Au/Ti/SiO_2_	1.6	0.14	-	[156]
	Ag/SiO_2_/TiN	1.3	3.3	-	[157]
	Ag/SP-GaO_x_/SP-AlO_x_/ITO	0.64	0.15	-	[158]

**Table 4:** Device performances of polymer-based memristors compared with those of other material based memristors.

Materials	Device Architectures	Thickness (nm)	Switching Time	LTP Pulse Voltage (V)	LTD Pulse Voltage (V)	Energy Consumption	Ref.
Perovskite	Pt/CoO/SrTiO_3_: Nb/Pt	50	2 ms	0.8	−0.8	677 nJ	[160]
	Au/PZT/LSMO	4	100 ns	2.2	−2.5	22 aJ	[161]
	Ag/TiO_2_: Ag/Pt	30	100 ns	0.5	−0.8	22.9 pJ	[162]
	Cr/a-MoO_3_/Au	11.5	200 ns	5.3	−3.5	2.34 pJ	[163]
Halide perovskite	Au/MAPbBr_3_/Au	1200	808 ms	0.2	−0.2	14.3 fJ	[164]
	Al/MAPBClBr_2_/Si: As	95.5	25 ms	0.5	−0.5	5.8 pJ	[165]
Polymer	Au/APP/ITO	112	100 ns	5	−5	384 pJ	[166]
	Ag/PVPy: Au@AgNPs/ITO	113.4–194.6	10 ms	5	−5	60 nJ	[167]
	Au/lignin/ITO	100	100 ms	0.7	−0.7	40 nJ	[168]

**Table 5:** Summarization of polymer memristor device for application in pattern and image recognition.

Structure	Non-Linearity (Potentiation)	Non-Linearity (Depression)	Applications (Accuracy)	Ref.
ITO/PEDOT: PSS (3% ZnO NPs)/ITO	0.96	−0.33	Pattern recognition 92%	[95]
ITO/PEDOT: PSS/pentacene/Au	1.51	2.20	Pattern recognition (92.6%)	[105]
ITO/P3OT/Al	0.37	0.46	Pattern recognition (91%)	[128]
Cu/pV3D3/Pt	−1.47	1	Face recognition (91%) Pattern recognition (86%)	[129]
Au/DPDA/ITO	0.005	0.20	Pattern recognition (95%)	[146]
ITO/Li^++^-PEDOT: PSS/TaN	1.5	0.4	Face recognition (96%)	[158]
IT0/PTBZ-DPP/Au	0.937	−1.172	Pattern recognition 92.78%	[178]
Au/PMMA/Cs_3_Bi_2_I_9_/PMMA/Au	0.8	1.1	Pattern recognition (86%)	[179]
Au/PMMA/PEA_2_MA_4_Pb_5_I_16_/Au	0.07	0.1	Pattern recognition (96%)	[180]
Ag/CuTCPP/Pt	0.08	0.05	Pattern recognition 93.51%	[181]

## Data Availability

Data sharing is not applicable to this article as no new data were created or analyzed in this study.
